# Supplementary material for: Phase separation of PGL-3 driven by structured domains that oligomerize and interact with RGG motifs
Source: EMBO Rep. 2026 Mar 20;27(8):2061–87. doi: 10.1038/s44319-026-00730-7 (PMC13121748; doi:10.1038/s44319-026-00730-7)
Supplement: Supplementary file 1 — Appendix [file 44319_2026_730_MOESM1_ESM.pdf]

**Appendix**

# Phase separation of PGL-3 driven by structured domains that oligomerize and interact with RGG motifs

Rimpei Kuroiwa, Piyoosh Sharma, Andrea Putnam, Stephen D. Fried, Geraldine Seydoux

Geraldine Seydoux
Email: gseydoux@jhmi.edu

Table of contents

Appendix Figure S1 ---------- 2
Appendix Figure S2 ---------- 3
Appendix Figure S3 ---------- 4
Appendix Figure S4 ---------- 5
Appendix Figure S5 ---------- 6
Appendix Figure S6 ---------- 7
Appendix Figure S7 ---------- 8
Appendix Table S1 ---------- 9
Appendix References ---------- 10

**A**

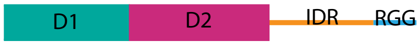


```
WATER
# Length: 748
# Identity:     444/748 (59.4%)
# Similarity:   543/748 (72.6%)
# Gaps:          75/748 (10.0%)

                        10        20        30        40        50        60        70        80        90
PGL-1           1 MEANKREIVDFGGLRSYFFPNLAHYITKNDEELFNNTSQANKLAAFVLGASKDAPGDEDILEMILPNDANAAVIAAGMDVCLLLGDKFRPKFDAAAEKLS    100
                  ||||||:||:..|::||||:||||:..|||.|.||.:|||||||||||:...|.:|:|.|||||||::|.|:||||||||:|||.||||||:.|||||
PGL-3           1 MEANKRQIVEVDGIKSYFFPHLAHYLASNDELLVNNIAQANKLAAFVLGATDKRPSNEEIAEMILPNDSSAYVLAAGMDVCLILGDDFRPKFDSGAEKLS    100
                        10        20        30        40        50        60        70        80        90

                        10        20        30        40        50        60        70        80        90
PGL-1         101 GLGHAHDLVSVIDDDKKLGMLARKAKLKKTEDAKILQALLKVIAIDDAAEKFVELTELVSQLDLDFDVYVLTKILGLISEETSDEVDIIRDNVVNAFDSC    200
                  .||.||||..:|||:||:.|||||.||||:.||||||.||||:..::|.||||||:||.|.||||||||||.|:||..|||..:|::||||||.:||::|
PGL-3         101 QLGQAHDLAPIIDDEKKISMLARKTKLKKSNDAKILQVLLKVLGAEEAEEKFVELSELSSALDLDFDVYVLAKLLGFASEELQEEIEIIRDNVTDAFEAC    200
                        10        20        30        40        50        60        70        80        90

                        10        20        30        40        50        60        70        80        90
PGL-1         201 KPLLKQLMLDGPKSEPADPFISLLMDPLEESVGKVVNHIAQLFEEASKNEGDESLVLRSQLGYQLFFLIVRSLADGKREVSKKILSGIPTSVRAEVFPGL    300
                  ||||:||::|||.:..|||..||:.|.|||:.|.|:||...||||..|.||||||:|||||||.||:|||||||||:.|:.|.|.:|:||||||||||
PGL-3         201 KPLLKKLMIEGPKIDSVDPFTQLLLTPQEESIEKAVSHIVARFEEASAVEDDESLVLKSQLGYQLIFLVVRSLADGKRDASRTIQSLMPSSVRAEVFPGL    300
                        10        20        30        40        50        60        70        80        90

                        10        20        30        40        50        60        70        80        90
PGL-1         301 QRSVYKSAVFLGNHIIQVLLGSKKSFEDWDVVGVAKDLESAWKRRAIAELIKKFQVSILEQCFDKPVPLIPQSPLNNDAVIDNVNKALQFALWLTEFYGS    400
                  ||||:||||||.:|||||.|||.||||||..||:|:||||.|:||||||||:|||::|:|||||.:|:||:|||.|||:.||:|||.|||||||:||||||
PGL-3         301 QRSVFKSAVFLASHIIQVFLGSMKSFEDWAFVGLAEDLESTWRRRAIAELLKKFRISVLEQCFSQPIPLLPQSELNNETVIENVNNALQFALWITEFYGS    400
                        10        20        30        40        50        60        70        80        90

                        10        20        30        40        50        60        70        80        90
PGL-1         401 ENETEALGELRFLDSTSKNLLVDSFKKFVQGINSKTHVTRIVESLEKCCLSDTPSGRKSNVQPSTSQQQDSAYTKEEMTTVHNTYSVNTKAQVLNGLSDT    500
                  |:|.::|.:|:||...||||||||||||.||::||.||.||:|||||          .|:.:||.        |.::.||.:...:|:|.|||
PGL-3         401 ESEKKSLNQLQFLSPKSKNLLVDSFKKFAQGLDSKDHVNRIIESLEK----------SSSSEPSA--------TAKQTTTSNGPTTVSTAAQV-------    475
                        10        20        30        40        48                  60        70

                        10        20        30        40        50        60        70        80        90
PGL-1         501 NSSGLLVDSKDSLSLQEISCDEVDSSTLLSSSRNIGEGVTVKAVDPVPEKVNDAQQQQTVNEIEMASDANQDTSSSASPEVAPSFSTDGWDSPTKSVALP    600
                       :...|...|.|.|.|:..|.:.:|:|::.|||.|||.|.|.:|||:|              ::.|.:|.|:|||   ..||:|||||||||||||
PGL-3         476 -----VTVEKMPFSRQTIPCEGTDLANVLNSAKIIGESVTVAAHDVIPEKLN--------------AEKNDNTPSTASP---VQFSSDGWDSPTKSVALP    553
                        80        90        00        10        20                       30         40        50

                        10          20        30        40        50        60        70        80        90
PGL-1         601 PGMQQIDEEETTVADKDST-----PQPQARAETAWGSGDATPMPLPAPTNQYKVSGFGEAKVAKGFGQFAPTSSAYGGGGGRGGYG---GGDRGGRGGYG    692
                  |.:..::||:    ::|:|     ||||.|..||||||||||:||..|.|:|||||||.|.||.||||||.::     |..|||.||   ||||||||.||
PGL-3         554 PKISTLEEEQ----EEDTTITKVSPQPQERTGTAWGSGDATPVPLATPVNEYKVSGFGAAPVASGFGQFASSN----GTSGRGSYGGGRGGDRGGRGAYG    645
                     60            70        80        90        00        10        20            30        40

                              00        10             20
PGL-1         693 GDRG------GRGGYGGGDRGGRGGYG-GDR----GRGGYGGRGGRGGF    730
                  ||||      |..||.||||||||.|| |.|    ||.|:.| |.|||
PGL-3         646 GDRGRGGSGDGSRGYRGGDRGGRGSYGEGSRGYQGGRAGFFG-GSRGGS    693
                      50        60        70        80        90
```

**B**

| | Identity | Similarity | Gap |
|---|---|---|---|
| FL | 444/748 (59.4%) | 543/748 (72.6%) | 75/748 (10.0%) |
| D1 | 145/212 (68.4%) | 178/212 (84.0%) | 0/212 ( 0.0%) |
| D2 | 169/243 (69.5%) | 205/243 (84.4%) | 0/243 ( 0.0%) |
| IDR | 90/208 (43.3%) | 122/208 (58.7%) | 38/208 (18.3%) |
| RGG | 43/71 (60.6%) | 44/71 (62.0%) | 15/71 (21.1%) |
| D1D2 | 309/447 (69.1%) | 375/447 (83.9%) | 0/447 ( 0.0%) |
| IDR-RGG | 133/283 (47.0%) | 166/283 (58.7%) | 57/283 (20.1%) |

**Appendix Figure S1.** (**A**) Alignment of PGL-1 and PGL-3, using EMBOSS Water (Madeira et al., 2024). (**B**) Table showing similarity and identity of protein domains between PGL-1 and PGL-3.

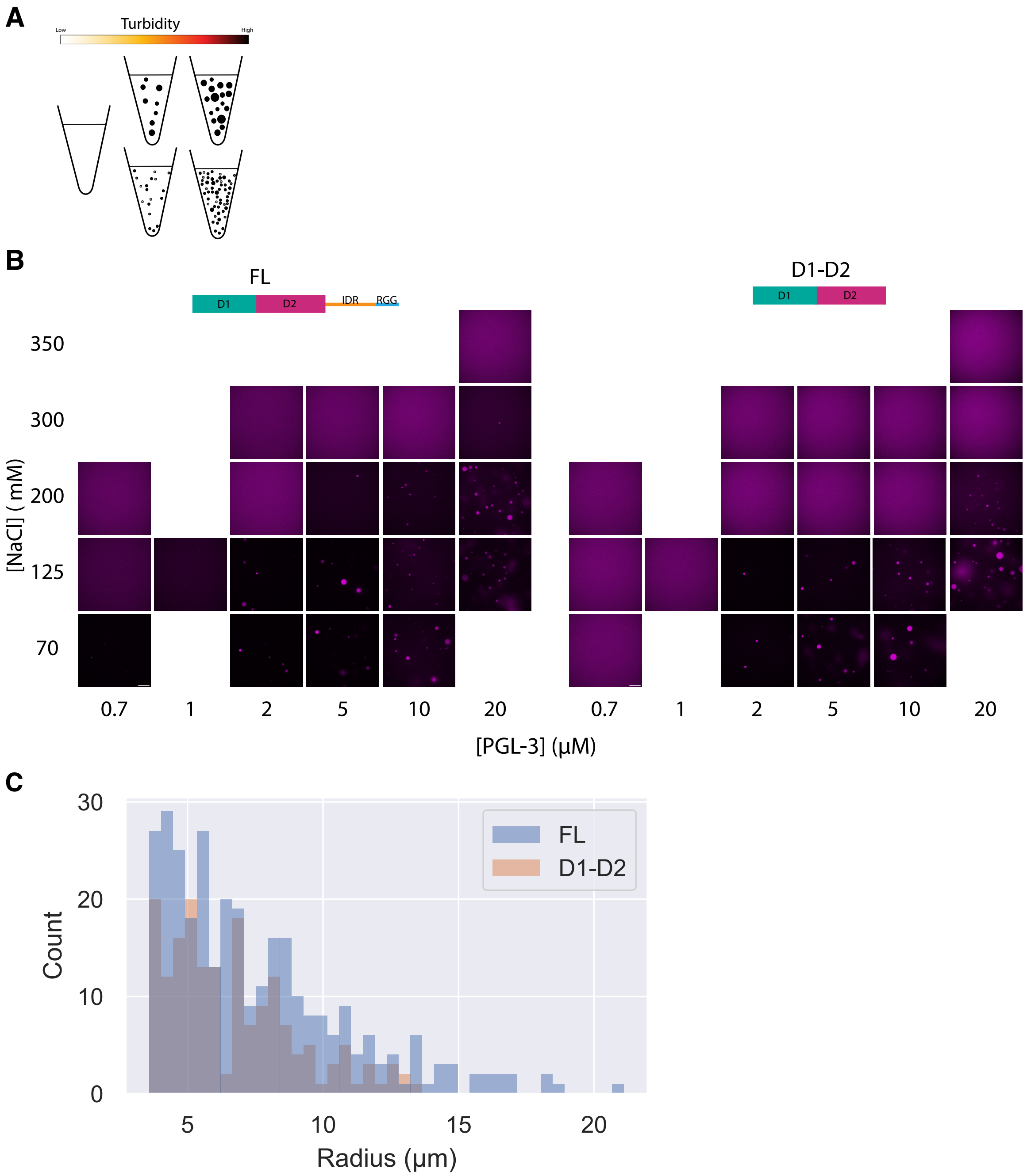


**Appendix Figure S2**. (A) Schematic describing the basis of turbidity measurement. Related to Fig. 2A. (B) Representative fluorescence micrographs at indicated protein and salt concentrations for full-length PGL-3 and D1-D2. Scale bar=20 μm. (C) Count histogram showing the size distribution of PGL-3 condensates. Related to Figure EV2B,C.

| | $C_{dil}$ (mean± 95% confidence interval) |
|---|---|
| **FL** | 0.27 ± 0.03 |
| **D1-D2** | 0.94 ± 0.03 |
| **D1-D2-RGG** | 0.51 ± 0.02 |
| **D1-D2-3x(GGGGS)-RGG** | 0.48 ± 0.01 |

**Appendix Figure S3.** Table showing $c_{dil}$ of PGL-3 constructs, related to Fig. 3B. Values show mean±95% confidence interval.

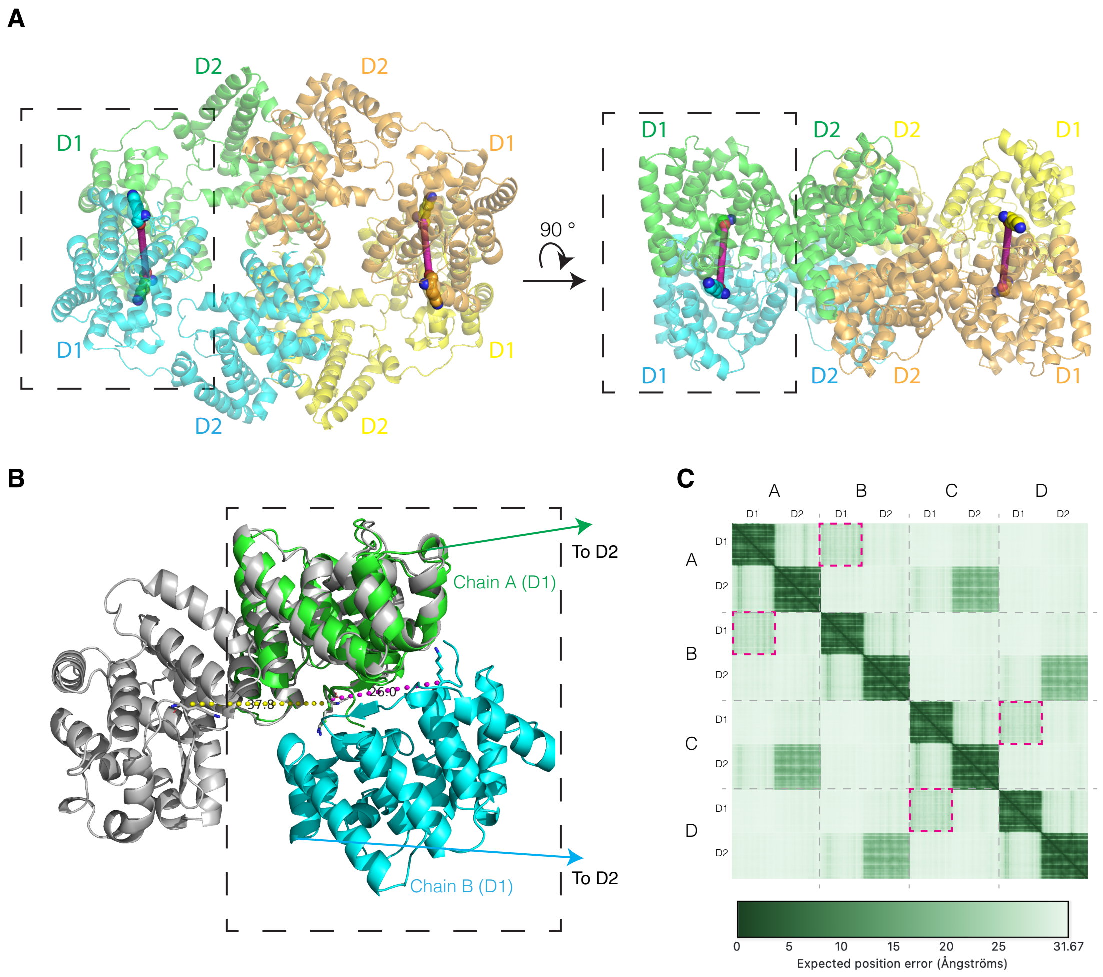


**Appendix Figure S4.** (A) Snapshot of an Alphafold3 model of a PGL-3(D1-D2) tetramer. The colors highlight the four D1-D2 chains (A:green, B:cyan, C:orange, D:yellow). Magenta lines show the Cα-Cα distance between K5 in D1 of chains A:B (26.0 Å) and C:D (26.7 Å). (B) Close-up snapshot of the D1:D1 interface from the model shown in the dotted square box in A. A dimer model (grey) is superimposed to highlight the use of different interfaces between the two models. The magenta line shows the Cα-Cα distance of the K5 dimer crosslink for chains A and B of the AF3 tetramer model, and the yellow line shows the same for the SWISS model based on the crystal structure reported in (Aoki et al., 2021). D2 domains and chains C and D are hidden for visualisation purposes. (C) Paired alignment errors (PAE) plot of the AF3 model shown in A. Magenta squares highlight the areas of D1 pairs that led to K5 dimer crosslink within 30Å.

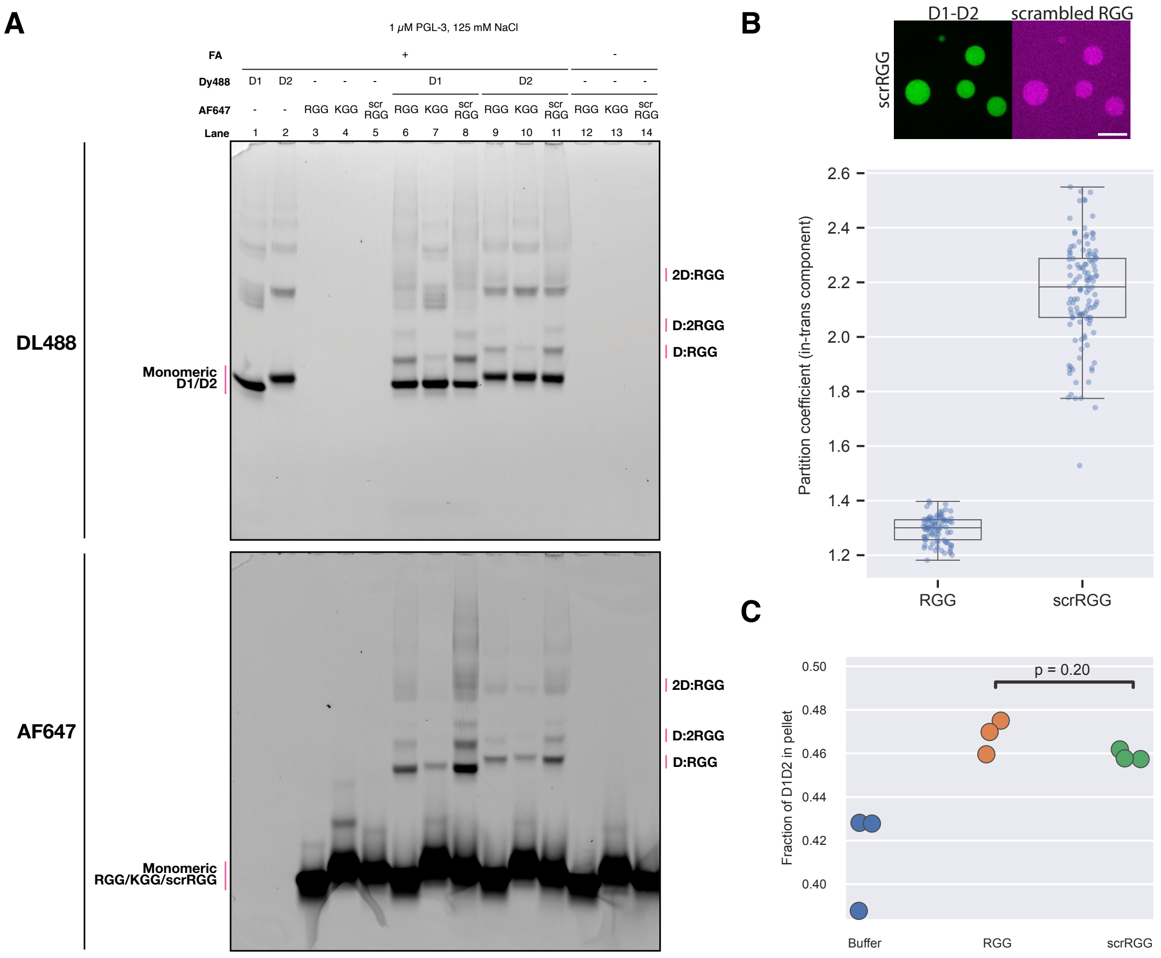


**Appendix Figure S5.** (**A**) Fluorescent images of SDS-PAGE gels showing crosslinked complexes of PGL-3 D1 or D2 with RGG, KGG or scrambled RGG (scrRGG) peptides. (**B**) Top: Photomicrograph showing partition of scrambled RGG (magenta) to D1-D2 condensates (green). Bottom: Graph showing the partition coefficient of scrambled RGG to D1-D2 condensates. In the overlaying box plots, lines inside boxes show median (Q2), box bounds are quartiles (Q1 and Q3), and whiskers show the range of data excluding outliers identified by the Turkey method. Scale bar = 10 μm. (**C**) Graph showing results from pelleting assay using scrambled RGG, where fractions of D1-D2 protein in pellet over total protein were calculated upon condensation of D1-D2 with the native or scrambled RGG and two phases were separated by centrifugation. P value by Wilcoxon rank-sum test. Technical replicates. N= 6.

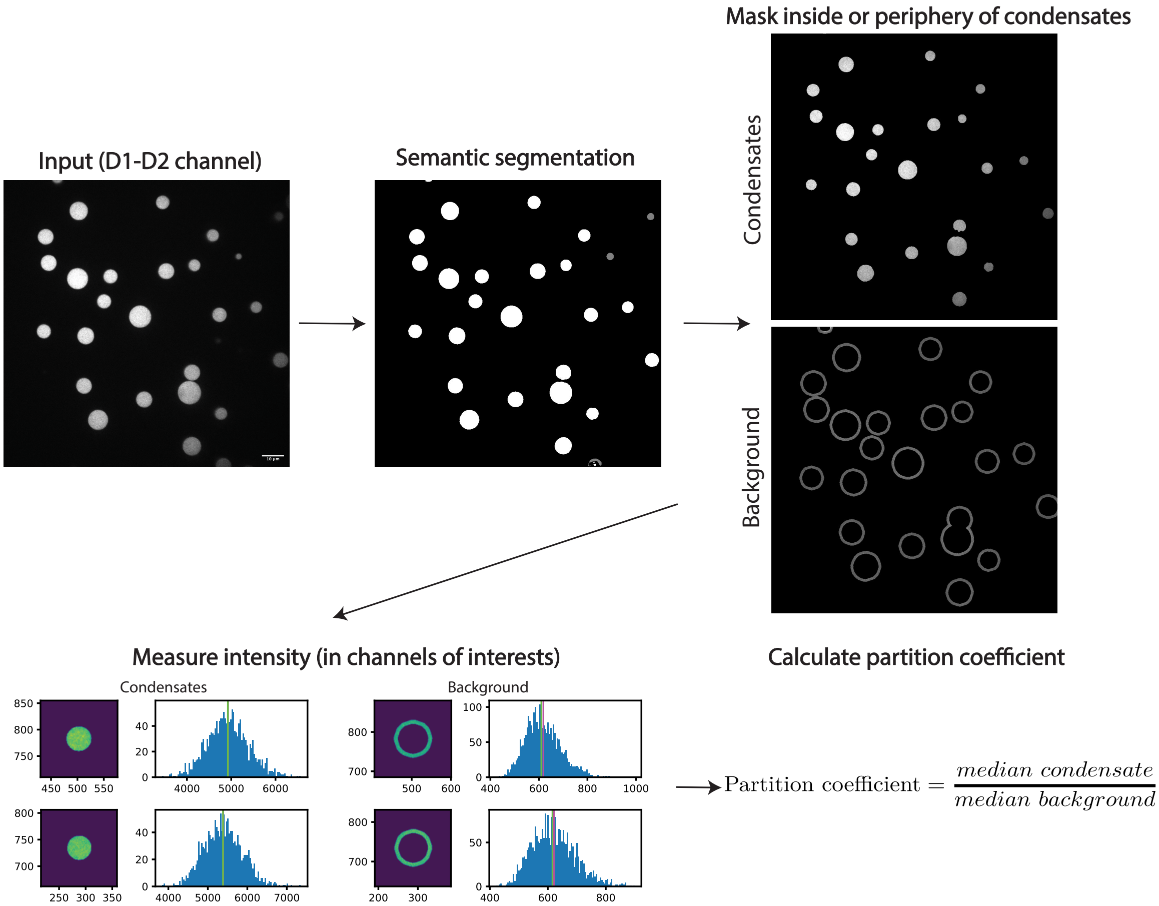


**Appendix Figure S6.** Schematics describing the algorithm for determining partition coefficient. Related to Figure 5B, C.

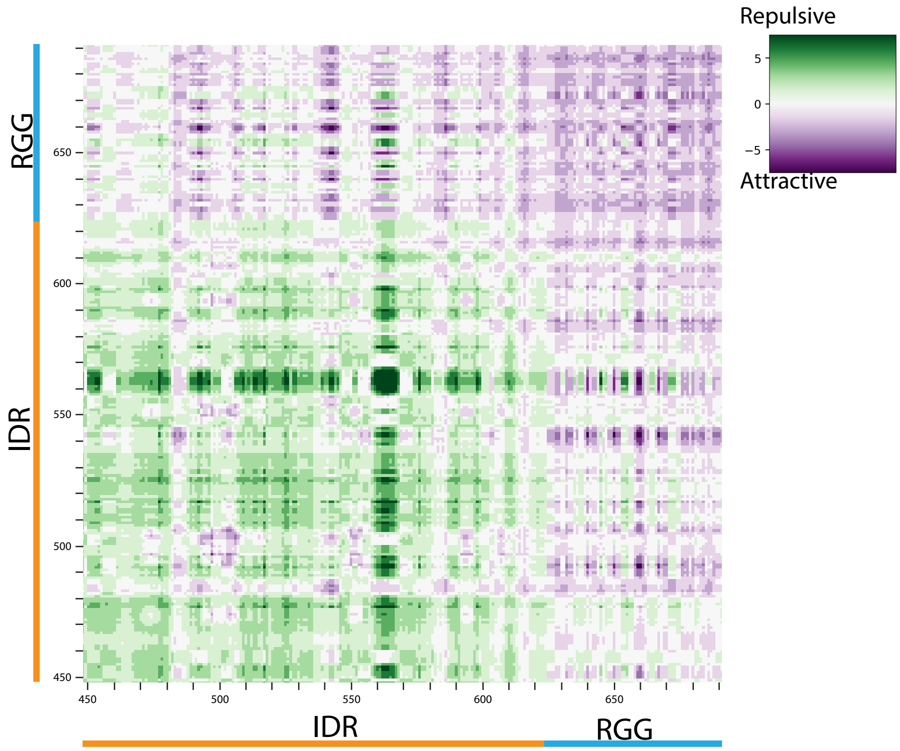


**Appendix Figure S7.** FINCHES (Ginell et al., 2025) intermap for IDR-RGG region of PGL-3. Green indicates repulsive and purple indicates attractive pairs.

**Appendix Table S1**. List of plasmids used to express PGL-3 and variants. All plasmids were prepared using the pMAL-c2X vector backbone.

| Plasmid | Construct | Tag (Removed by TEV cleavage during purification) | N-terminal linker (Remaining after TEV cleavage) | PGL-3 amino acids |
|---|---|---|---|---|
| pRK015 | PGL-3(FL) | 6xHis-MBP-6xHis-TEV | none | 1-693 |
| pRK049 | PGL-3(D1) | 6xHis-MBP-6xHis-TEV | none | 1-212 |
| pRK050 | PGL-3(D2) | 6xHis-MBP-6xHis-TEV | G | 205-447 |
| pRK011 | PGL-3(IDR) | MBP-6xHis-TEV | none | 448-622 |
| pRK036 | PGL-3(D1-D2) | 6xHis-MBP-6xHis-TEV | none | 1-447 |
| pRK042 | PGL-3(D1-D2-IDR) | 6xHis-MBP-6xHis-TEV | none | 1-622 |
| pRK082 | PGL-3(D1-D2-RGG) | 6xHis-MBP-6xHis-TEV | none | 1-447::623-693 |
| pRK084 | PGL-3(D1-D2-3(GGGGS)-RGG) | 6xHis-MBP-6xHis-TEV | none | 1-447::3x(GGGGS)::623-693 |
| pAAP35 | PGL-3(IDR-RGG) | MBP-6xHis-TEV | GAGL | 448-693 |
| pAAP36 | PGL-3(D1-IDR-RGG) | MBP-6xHis-TEV | GAGL | 1-212::448-693 |
| pAAP37 | PGL-3(D2-IDR-RGG) | MBP-6xHis-TEV | GAGL | 205-693 |
| pRK047 | PGL-3(FL)(R123E, K126E, K129E) | 6xHis-MBP-6xHis-TEV | none | 1-693(R123E, K126E, K129E) |

**Appendix References**


Aoki, S. T., Lynch, T. R., Crittenden, S. L., Bingman, C. A., Wickens, M., & Kimble, J. (2021). C. elegans germ granules require both assembly and localized regulators for mRNA repression. *Nature Communications 2021 12:1*, *12*(1), 1–14.

Ginell, G. M., Emenecker, R. J., Lotthammer, J. M., Keeley, A. T., Plassmeyer, S. P., Razo, N., Usher, E. T., Pelham, J. F., & Holehouse, A. S. (2025). Sequence-based prediction of intermolecular interactions driven by disordered regions. *Science*, *388*(6749).

Madeira, F., Madhusoodanan, N., Lee, J., Eusebi, A., Niewielska, A., Tivey, A. R. N., Lopez, R., & Butcher, S. (2024). The EMBL-EBI Job Dispatcher sequence analysis tools framework in 2024. *Nucleic Acids Research*, *52*(W1), W521–W525.
