# Supplementary figures and images for: Phase separation of PGL-3 driven by structured domains that oligomerize and interact with RGG motifs
Source: EMBO Rep. 2026 Mar 20;27(8):2061–87. doi: 10.1038/s44319-026-00730-7 (PMC13121748; doi:10.1038/s44319-026-00730-7)

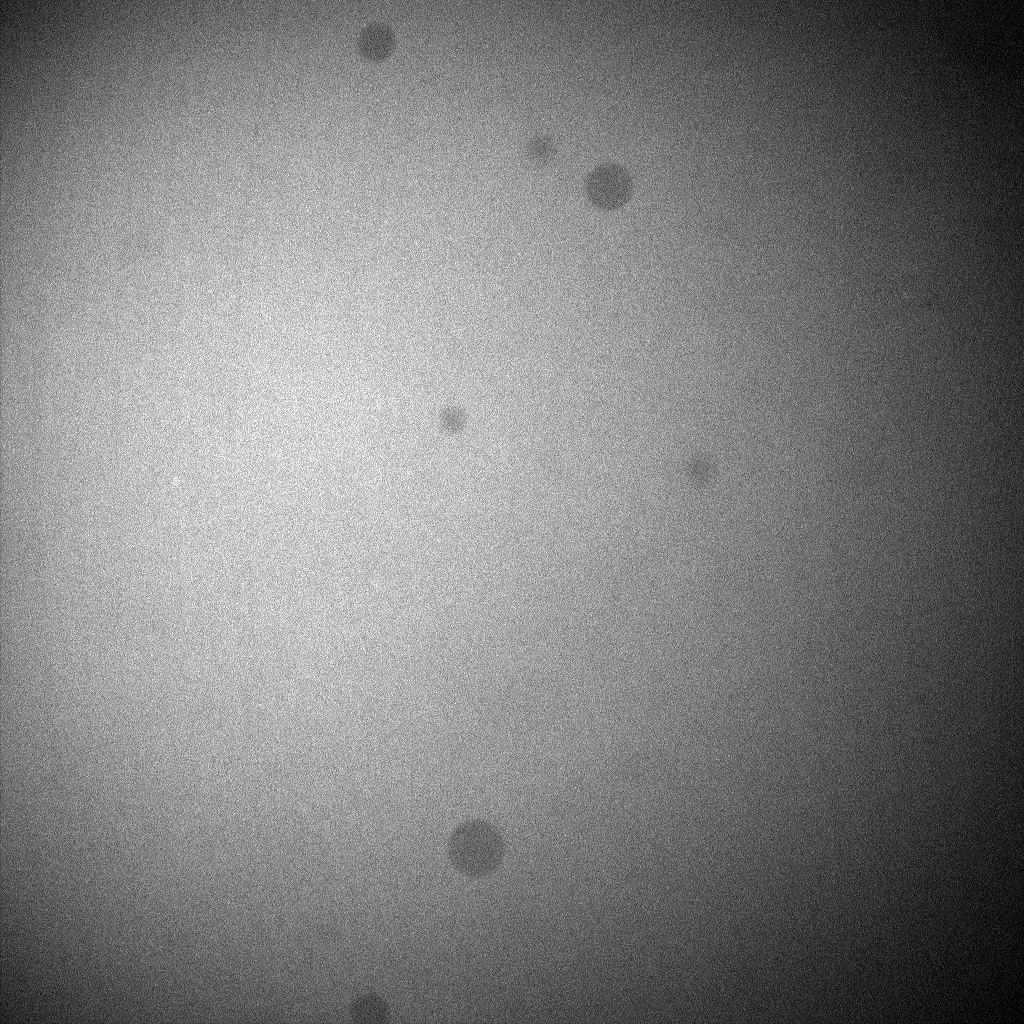

Supplement: Supplementary file 5 — Source data Fig. 1 [file 44319_2026_730_MOESM5_ESM.zip › Figure 1/1E/D1-D2, 260.tif]

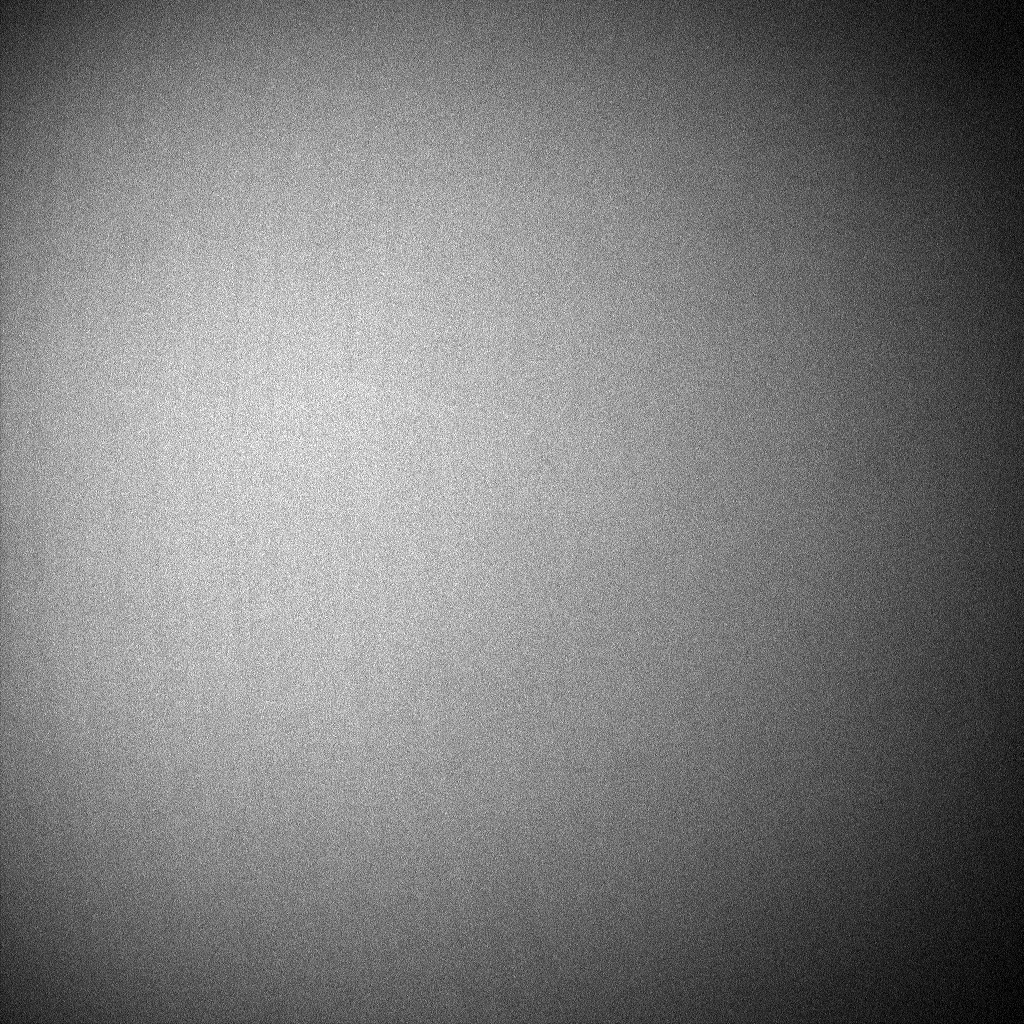

Supplement: Supplementary file 5 — Source data Fig. 1 [file 44319_2026_730_MOESM5_ESM.zip › Figure 1/1E/IDR-RGG, 260.tif]

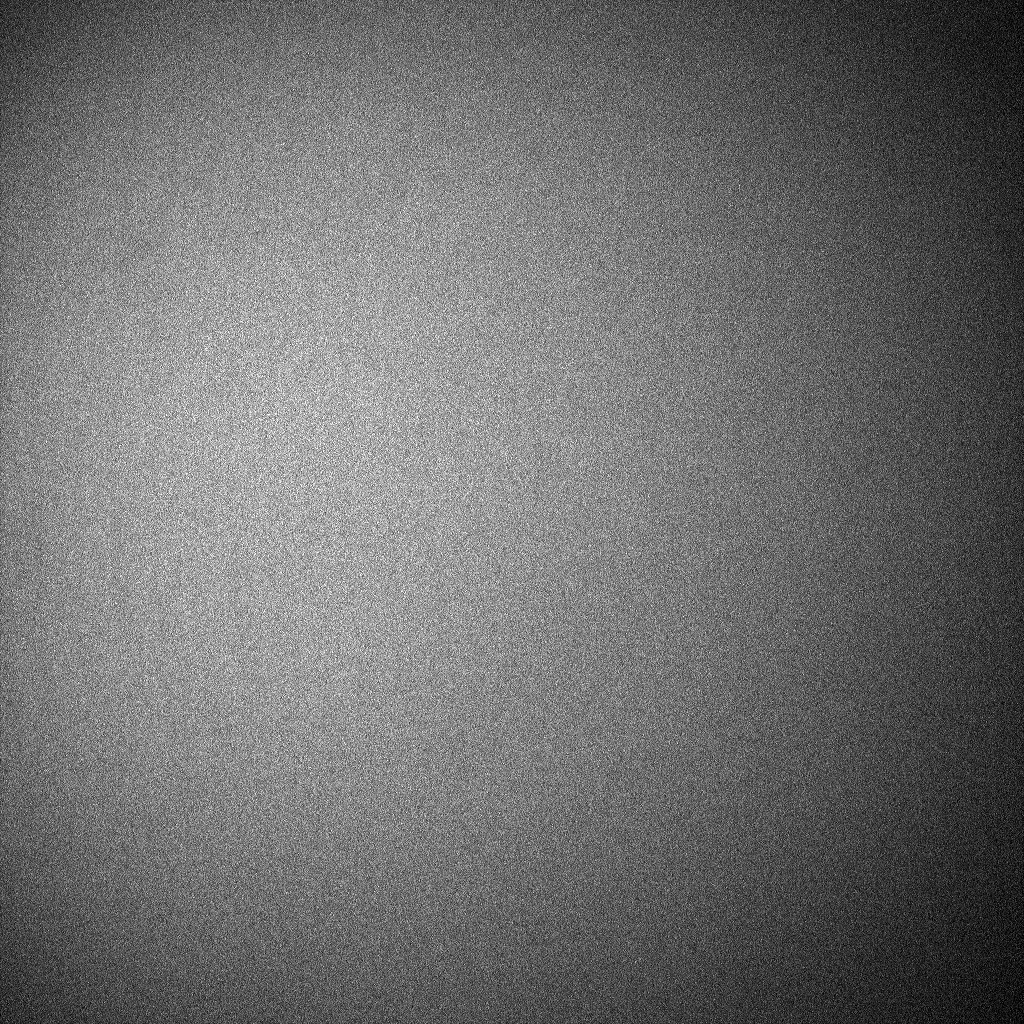

Supplement: Supplementary file 5 — Source data Fig. 1 [file 44319_2026_730_MOESM5_ESM.zip › Figure 1/1E/IDR-RGG, 80.tif]

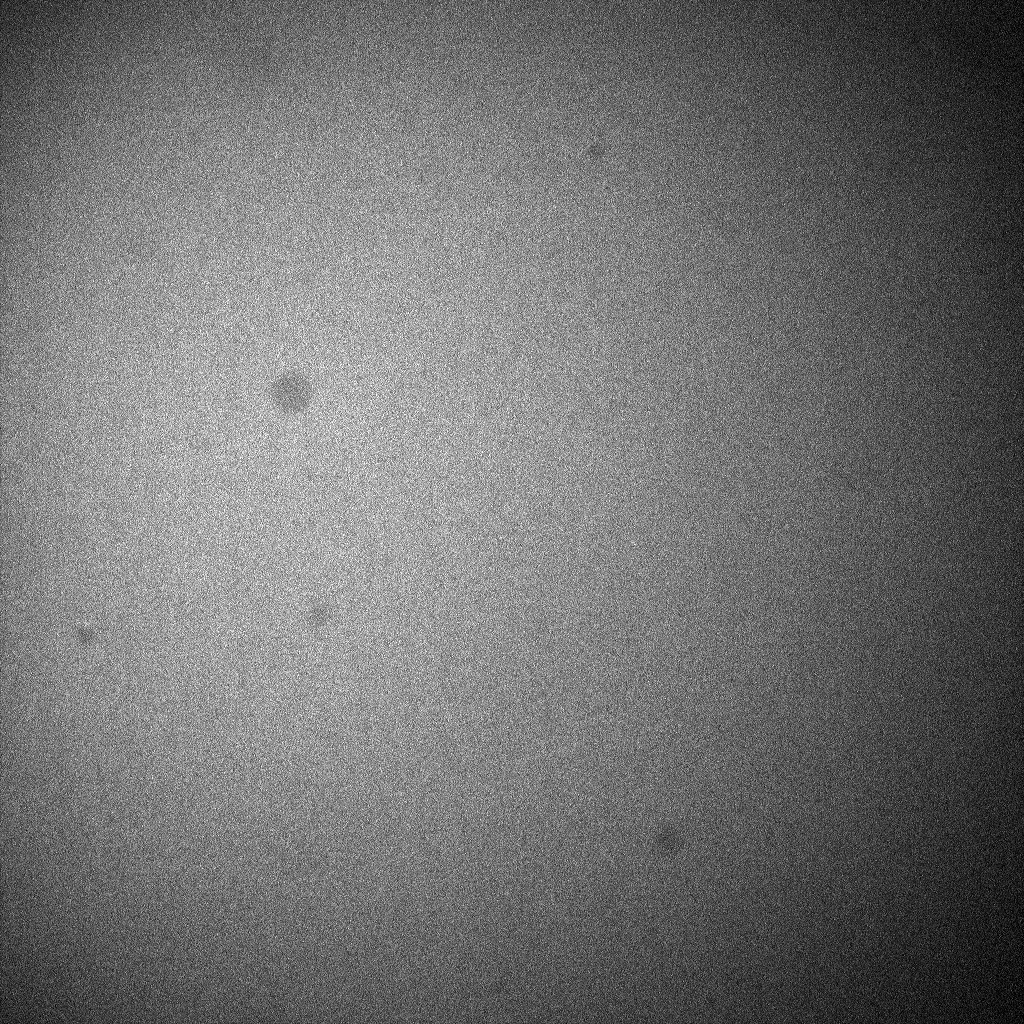

Supplement: Supplementary file 5 — Source data Fig. 1 [file 44319_2026_730_MOESM5_ESM.zip › Figure 1/1E/D1-D2, 80.tif]

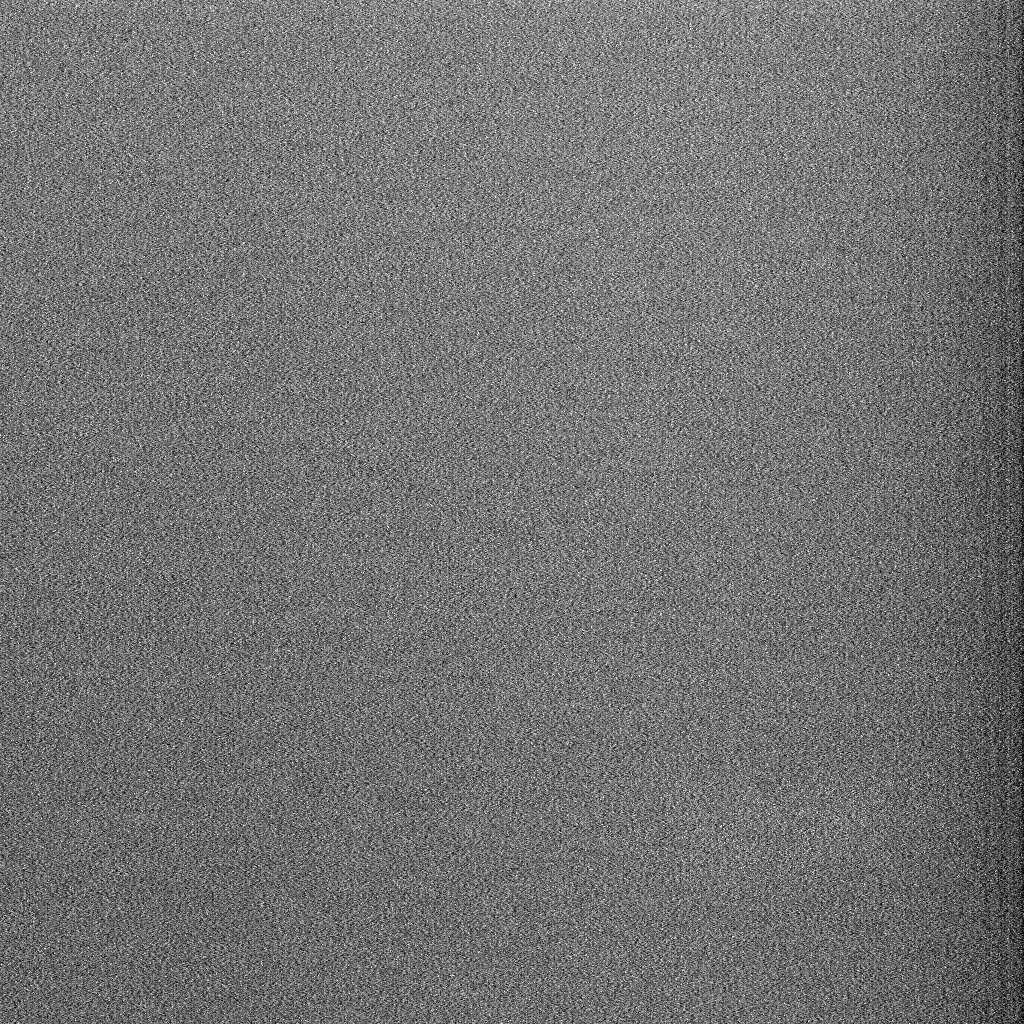

Supplement: Supplementary file 5 — Source data Fig. 1 [file 44319_2026_730_MOESM5_ESM.zip › Figure 1/1E/FL, 0.tif]

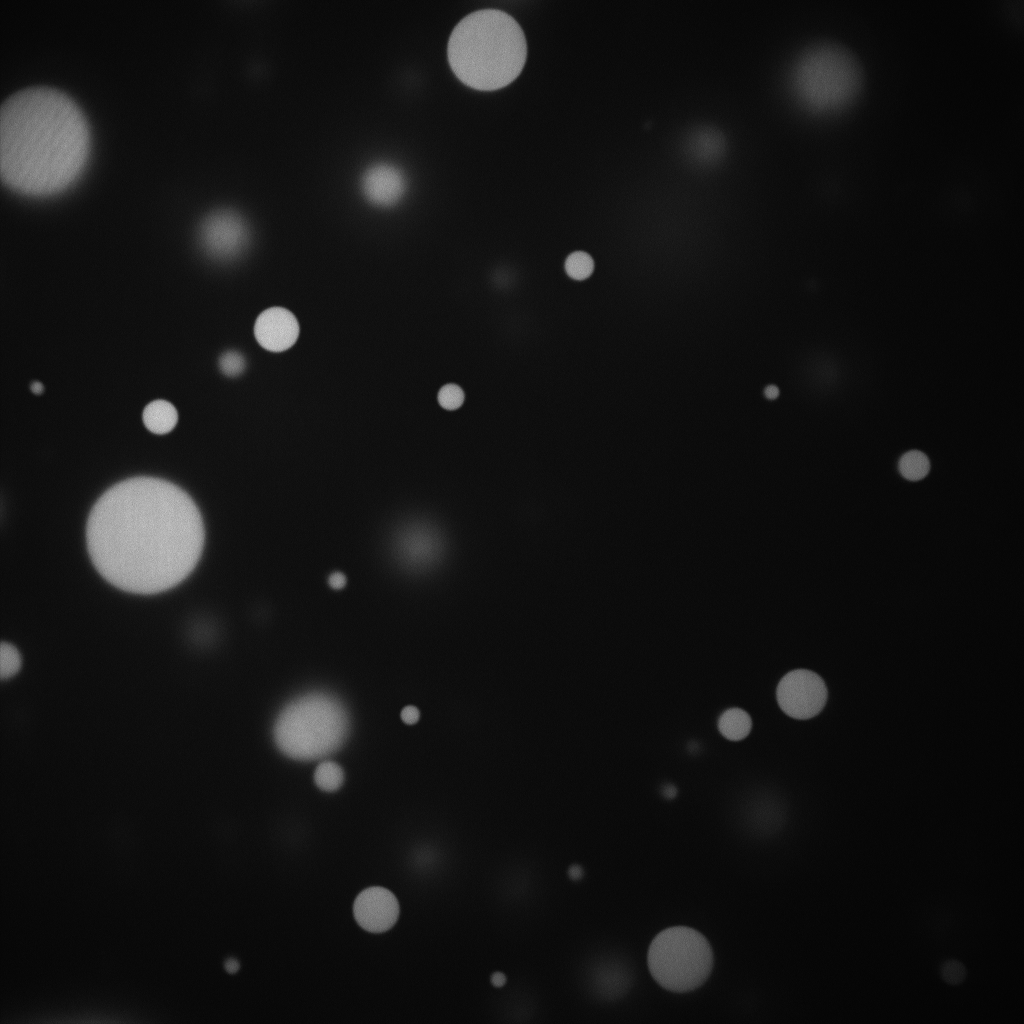

Supplement: Supplementary file 5 — Source data Fig. 1 [file 44319_2026_730_MOESM5_ESM.zip › Figure 1/1E/FL, 260.tif]

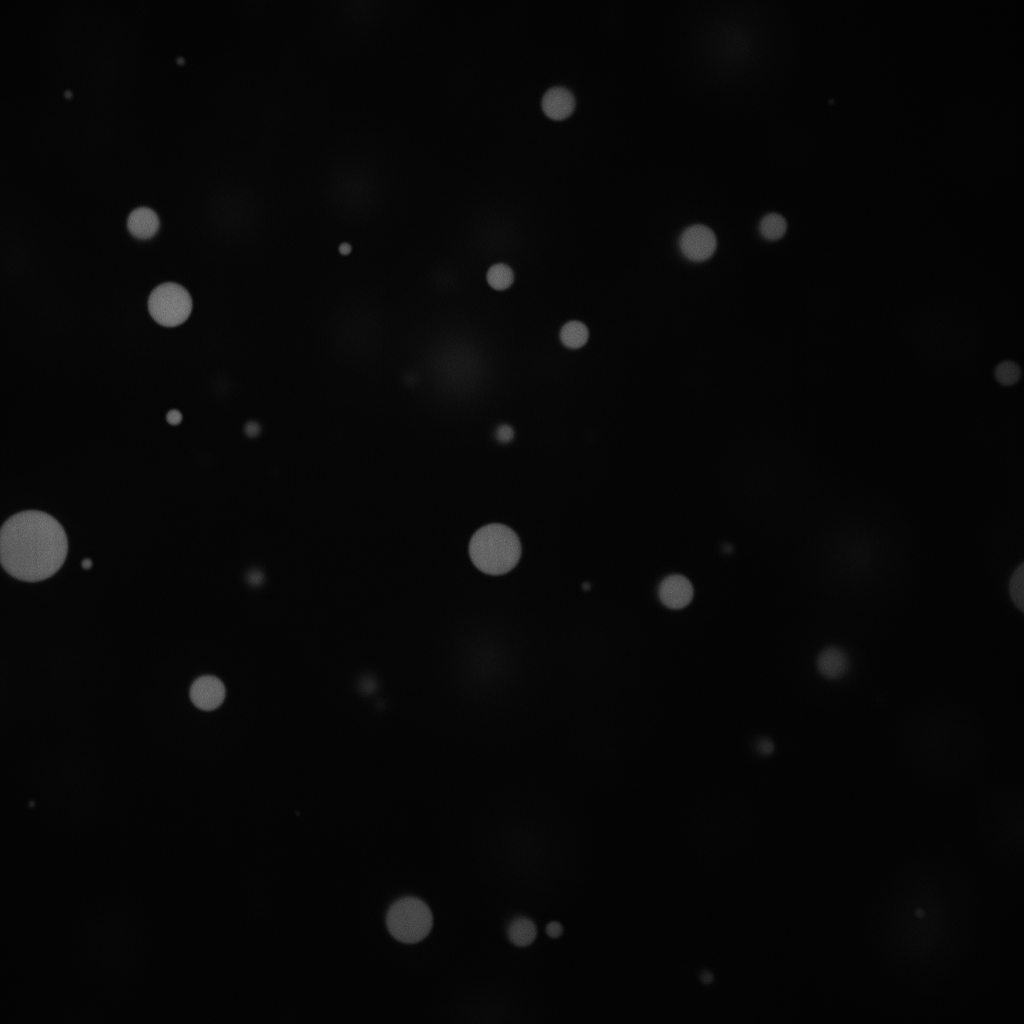

Supplement: Supplementary file 5 — Source data Fig. 1 [file 44319_2026_730_MOESM5_ESM.zip › Figure 1/1E/FL, 80.tif]

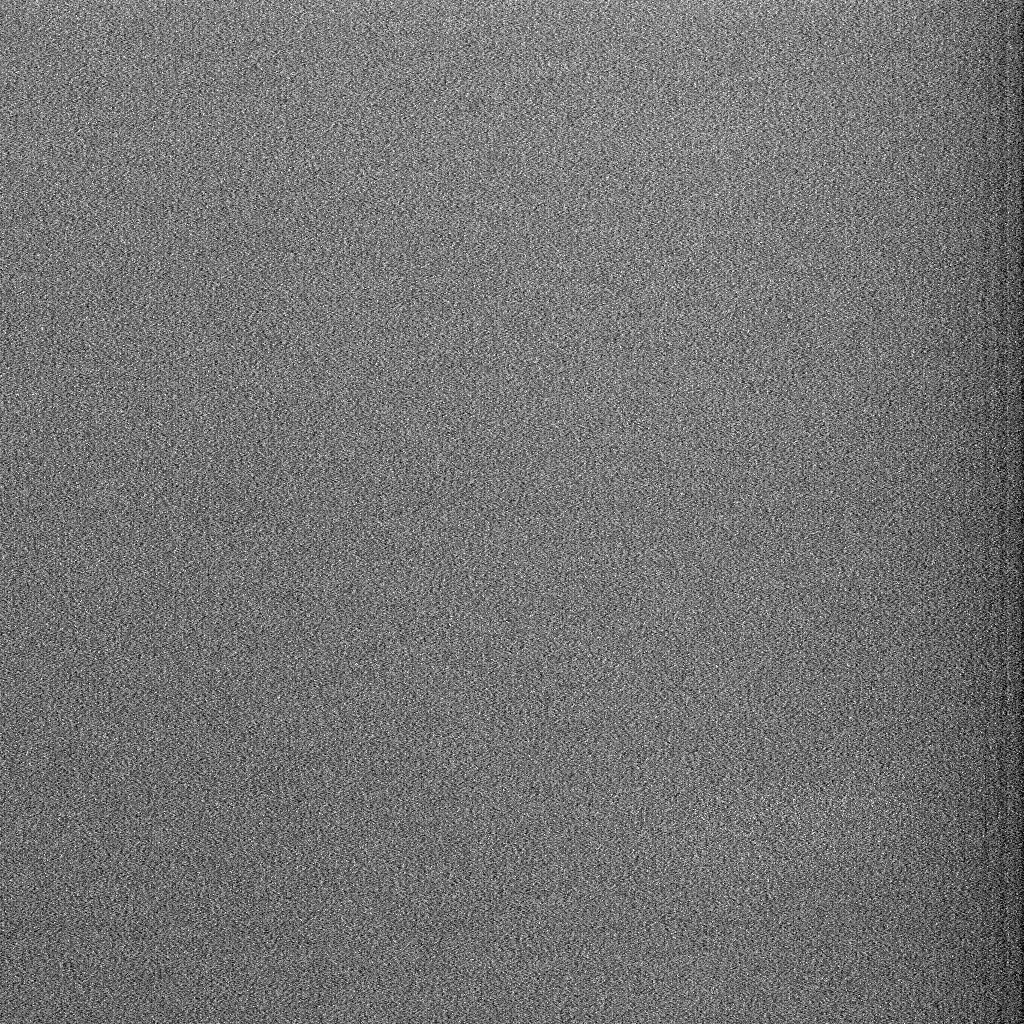

Supplement: Supplementary file 5 — Source data Fig. 1 [file 44319_2026_730_MOESM5_ESM.zip › Figure 1/1E/IDR-RGG, 0.tif]

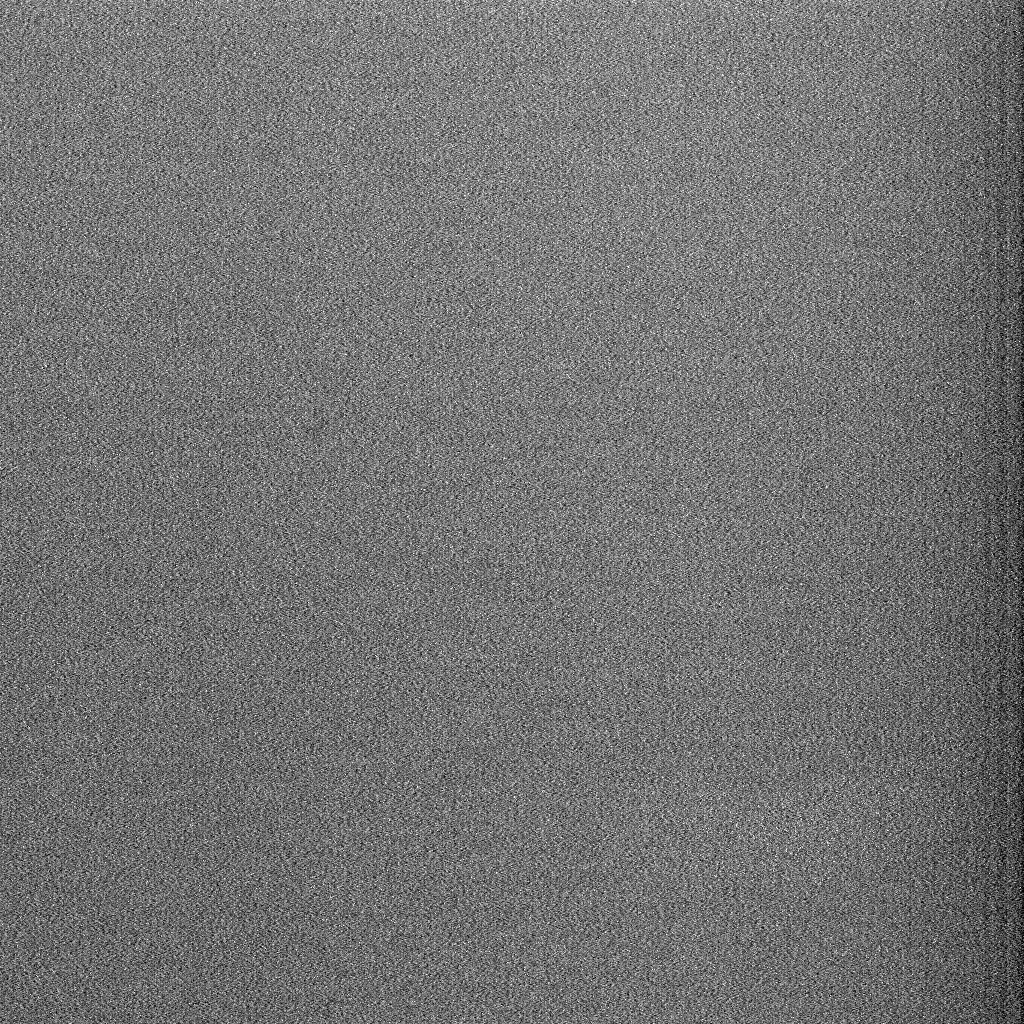

Supplement: Supplementary file 5 — Source data Fig. 1 [file 44319_2026_730_MOESM5_ESM.zip › Figure 1/1E/D1-D2, 0.tif]

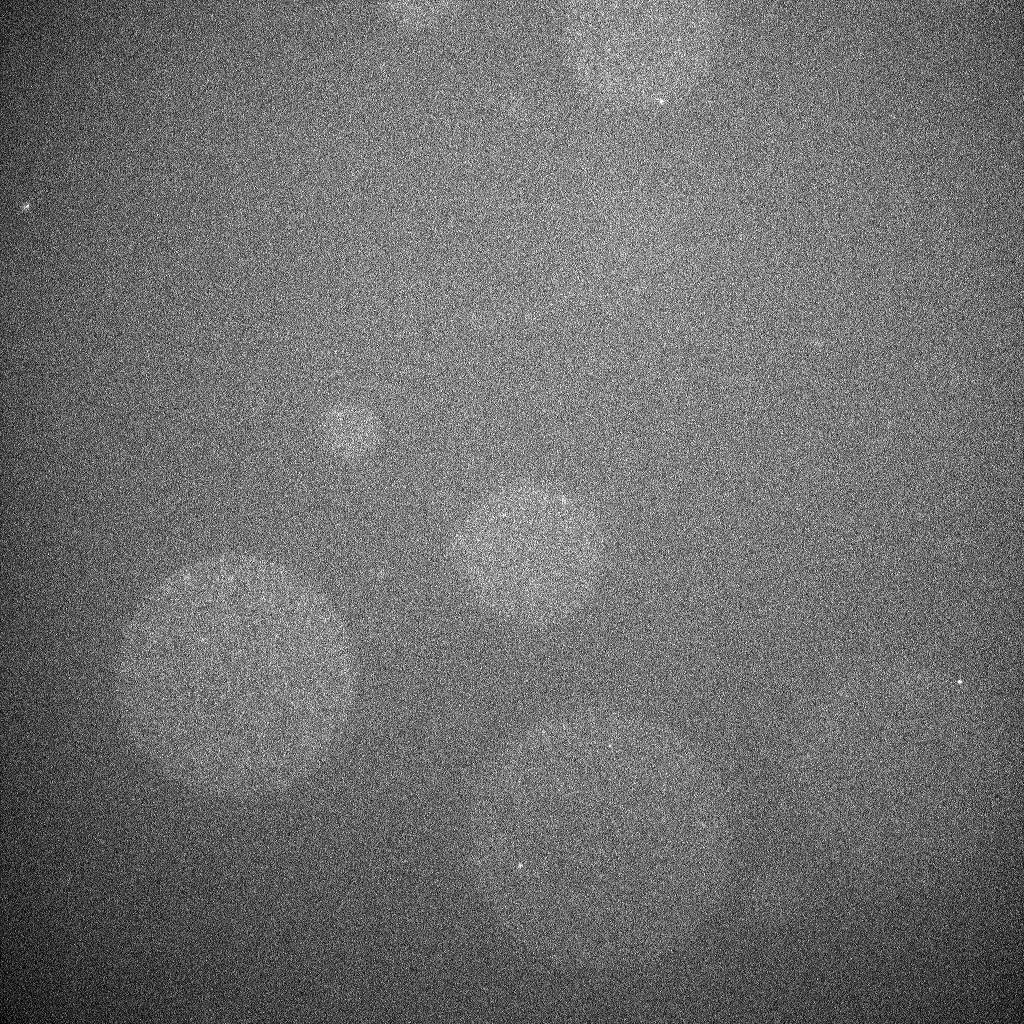

Supplement: Supplementary file 5 — Source data Fig. 1 [file 44319_2026_730_MOESM5_ESM.zip › Figure 1/1D/D1-D2-IDR.tif]

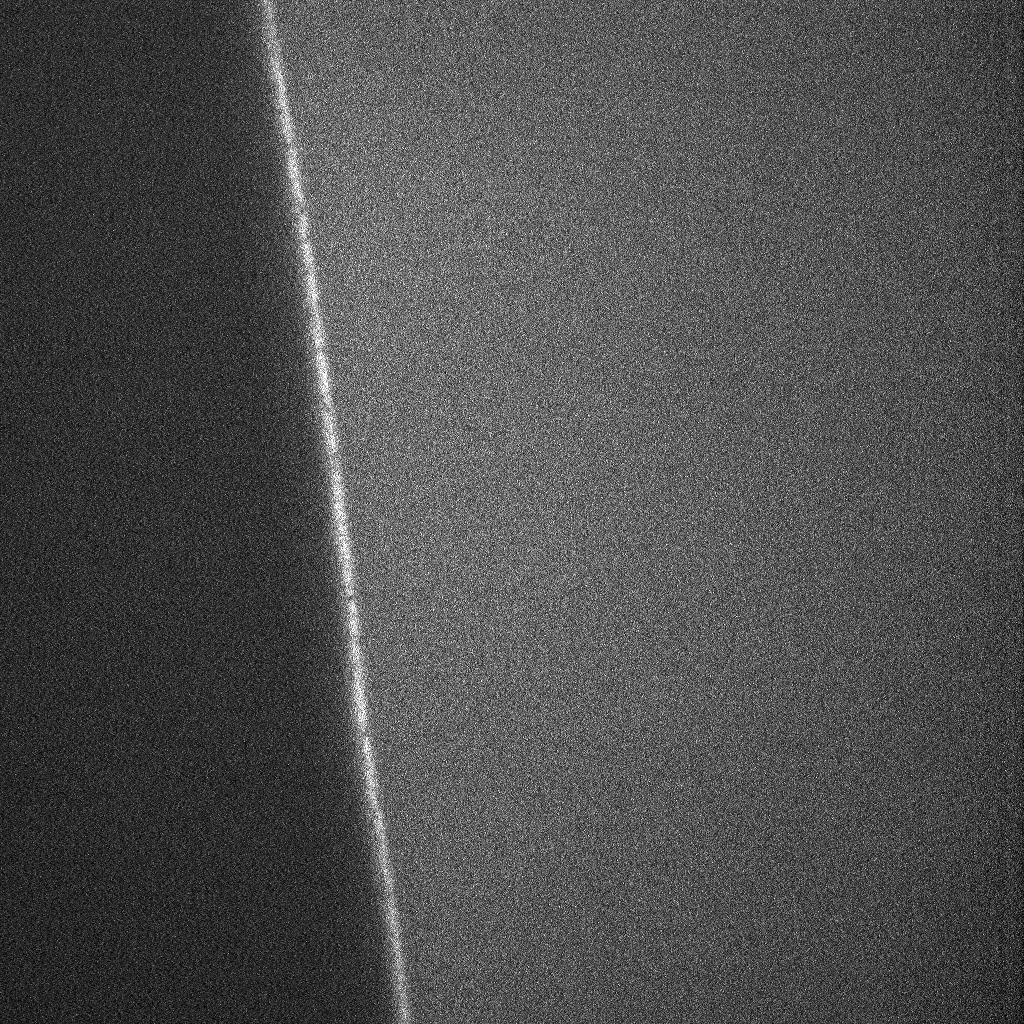

Supplement: Supplementary file 5 — Source data Fig. 1 [file 44319_2026_730_MOESM5_ESM.zip › Figure 1/1D/RGG.tif]

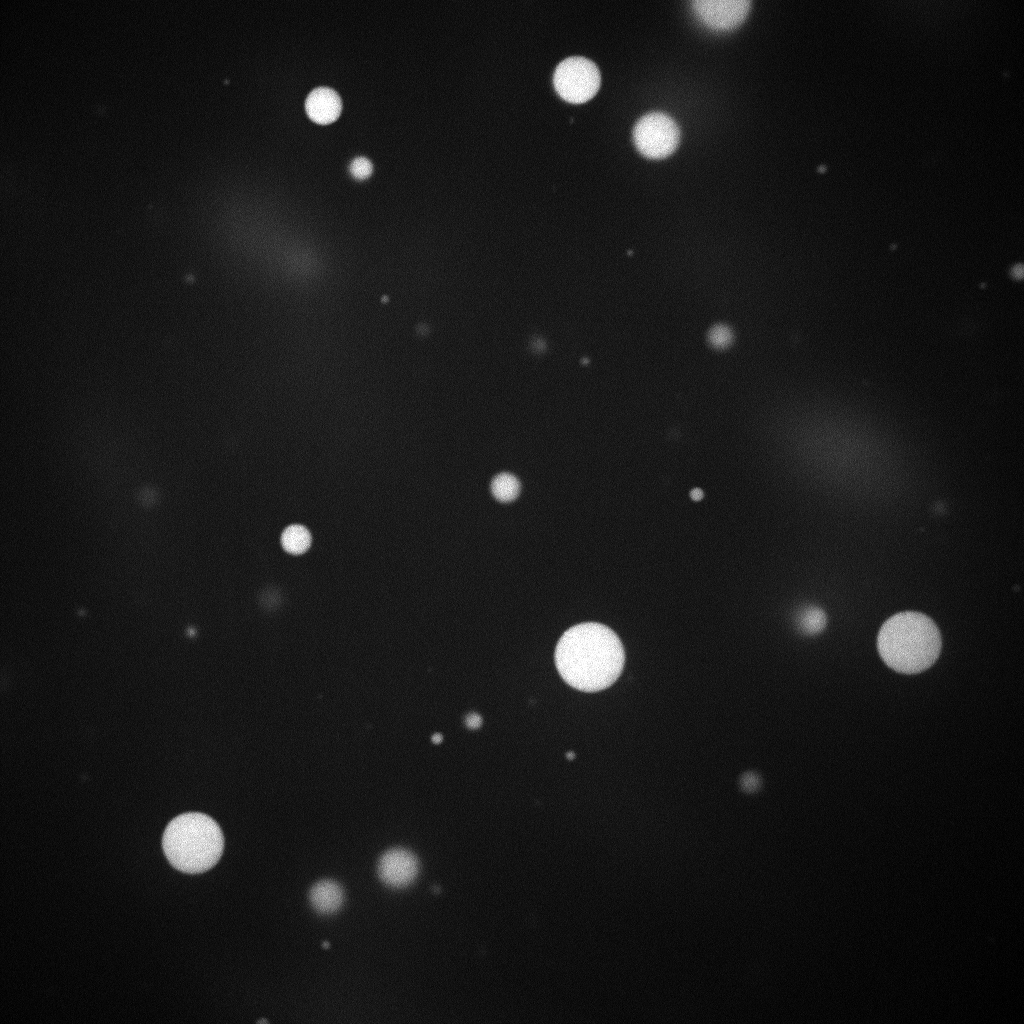

Supplement: Supplementary file 5 — Source data Fig. 1 [file 44319_2026_730_MOESM5_ESM.zip › Figure 1/1D/D1-D2.tif]

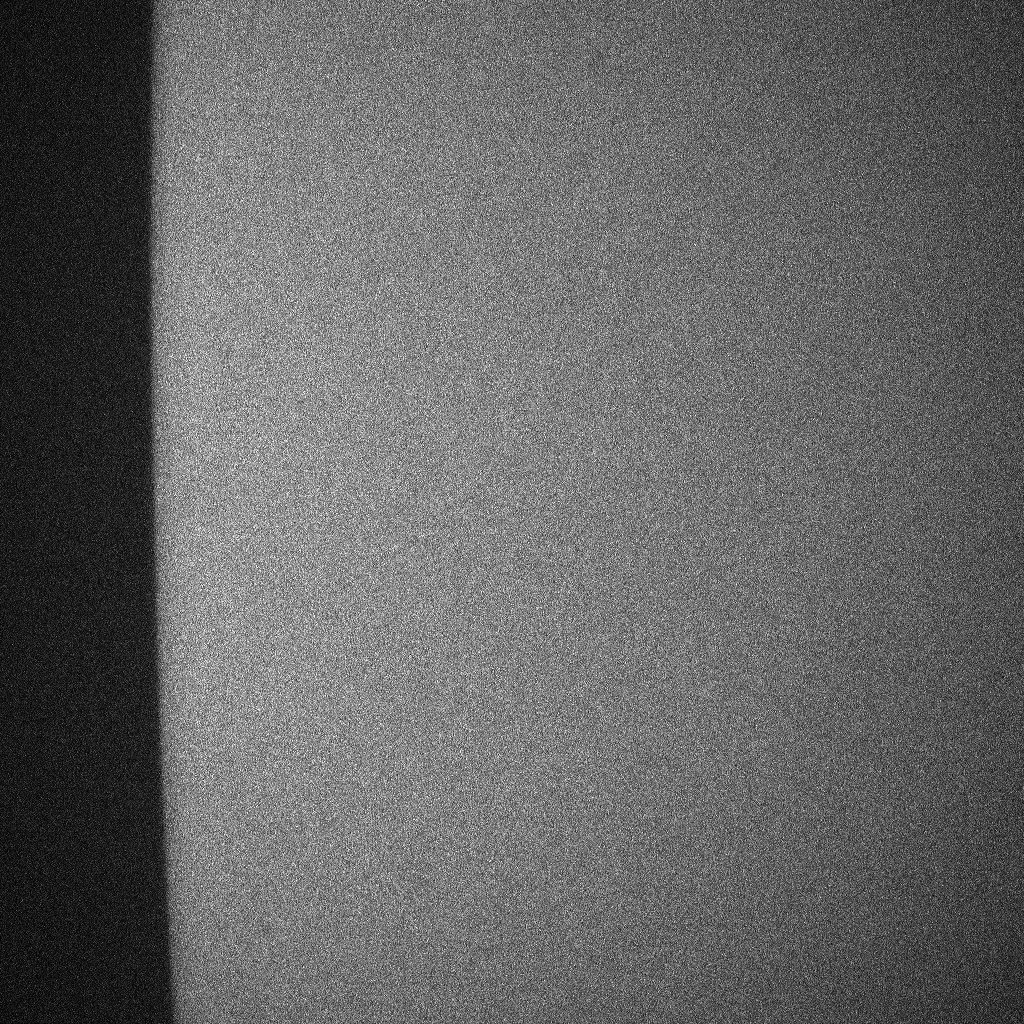

Supplement: Supplementary file 5 — Source data Fig. 1 [file 44319_2026_730_MOESM5_ESM.zip › Figure 1/1D/D2-IDR-RGG.tif]

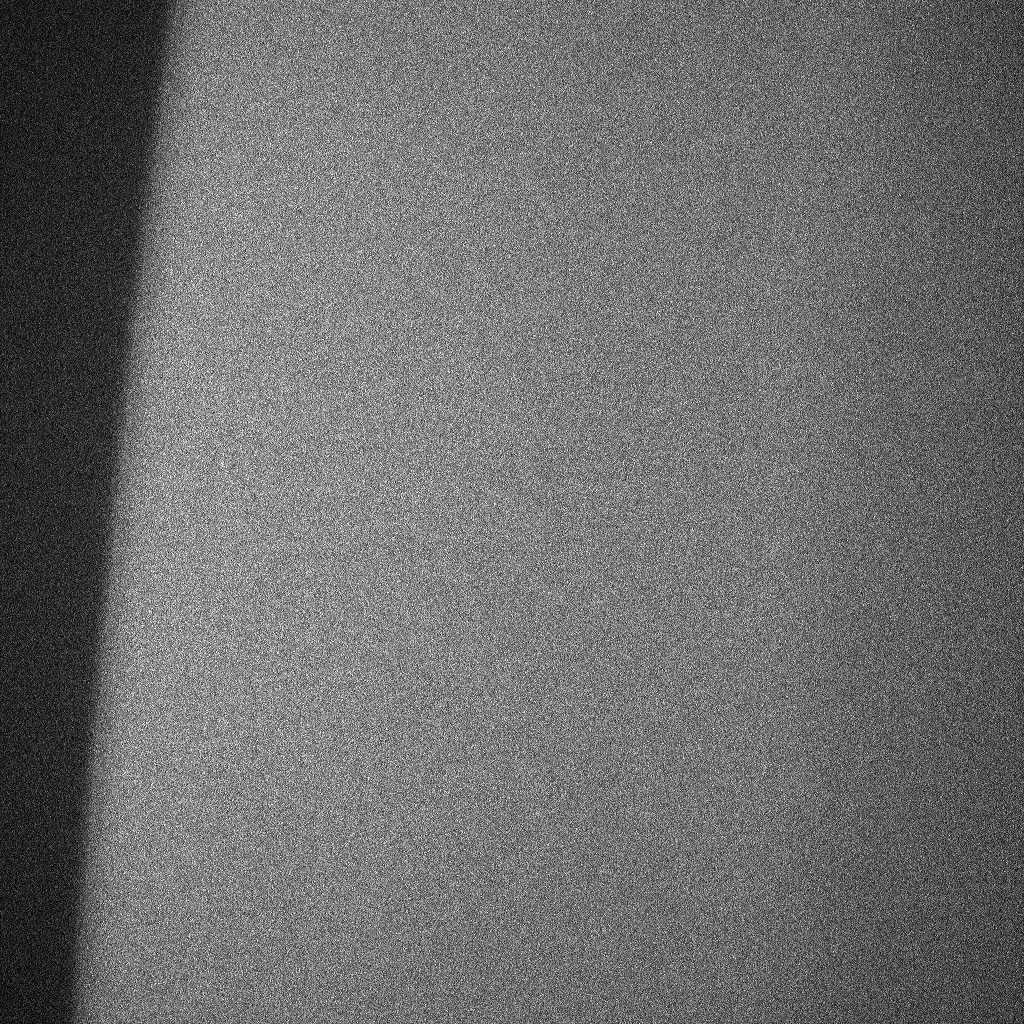

Supplement: Supplementary file 5 — Source data Fig. 1 [file 44319_2026_730_MOESM5_ESM.zip › Figure 1/1D/IDR.tif]

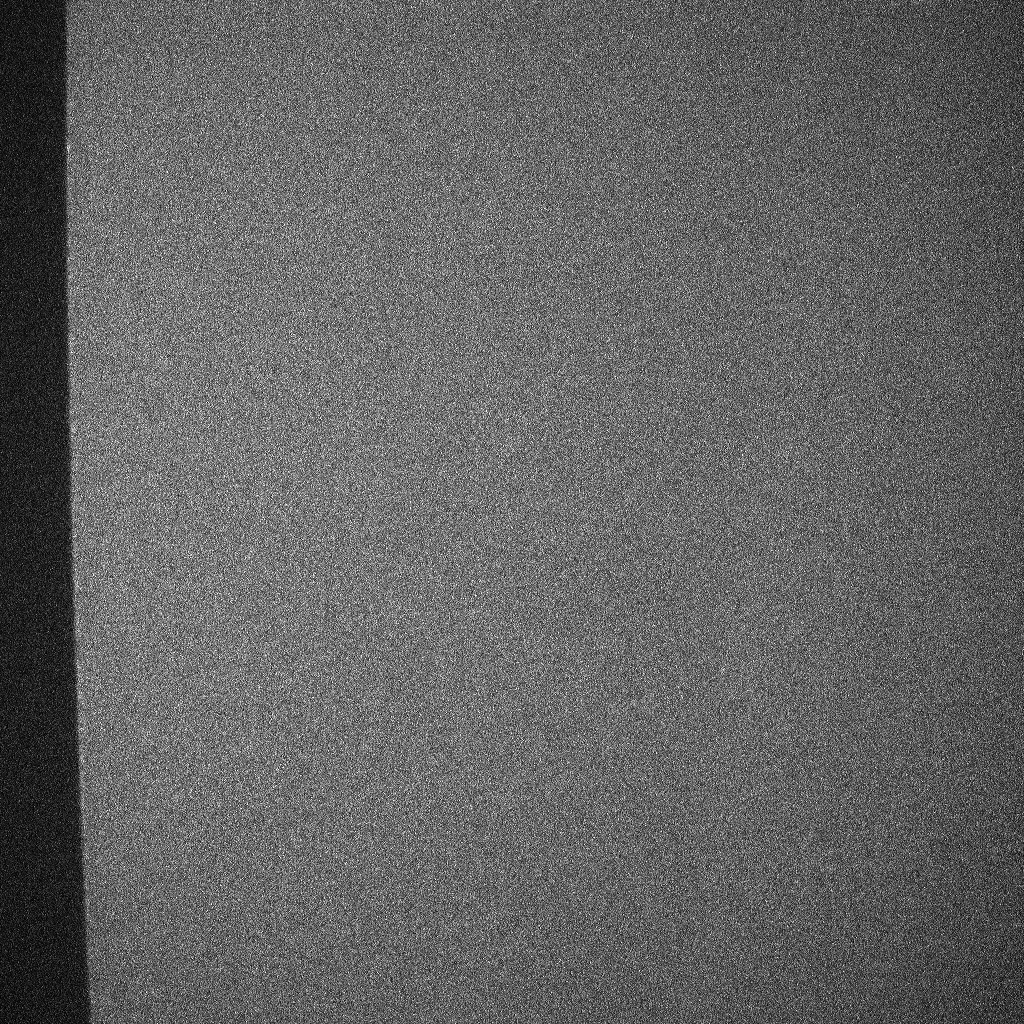

Supplement: Supplementary file 5 — Source data Fig. 1 [file 44319_2026_730_MOESM5_ESM.zip › Figure 1/1D/D1.tif]

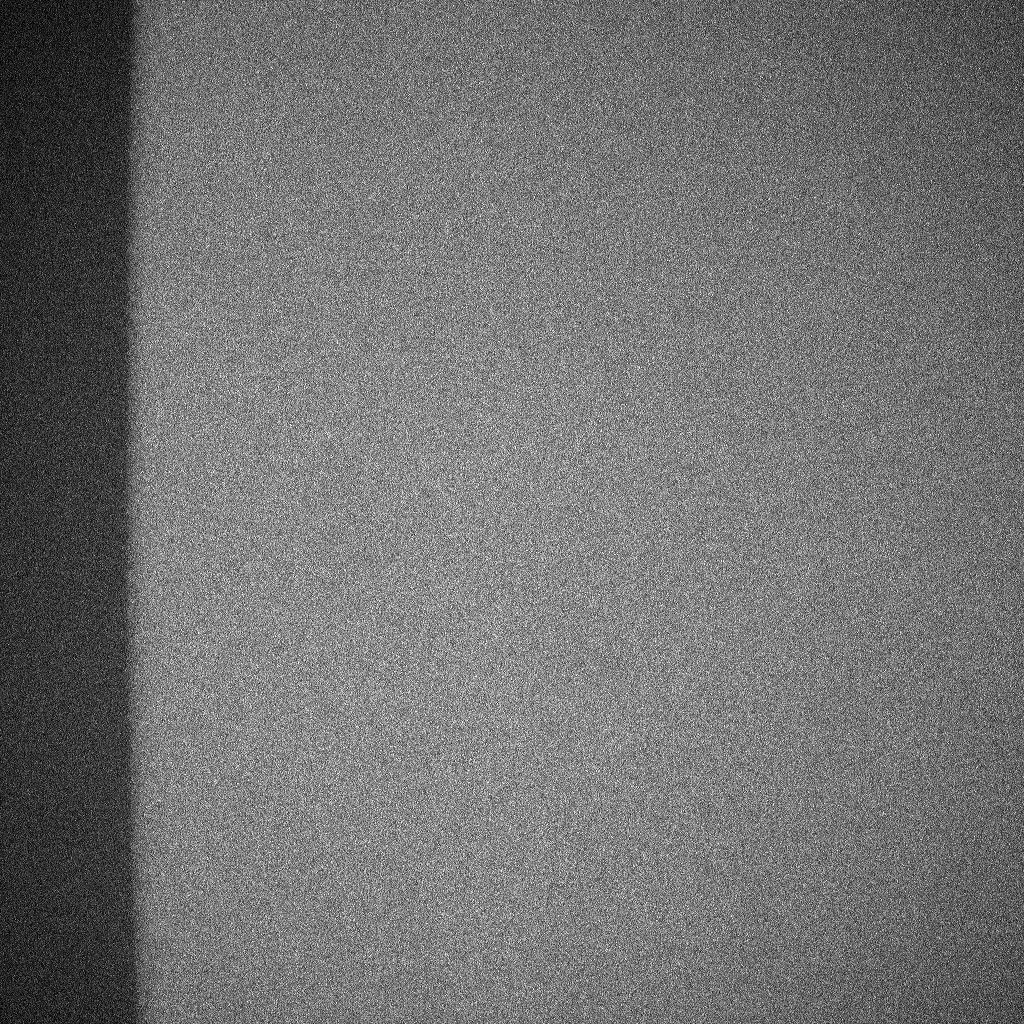

Supplement: Supplementary file 5 — Source data Fig. 1 [file 44319_2026_730_MOESM5_ESM.zip › Figure 1/1D/IDR-RGG.tif]

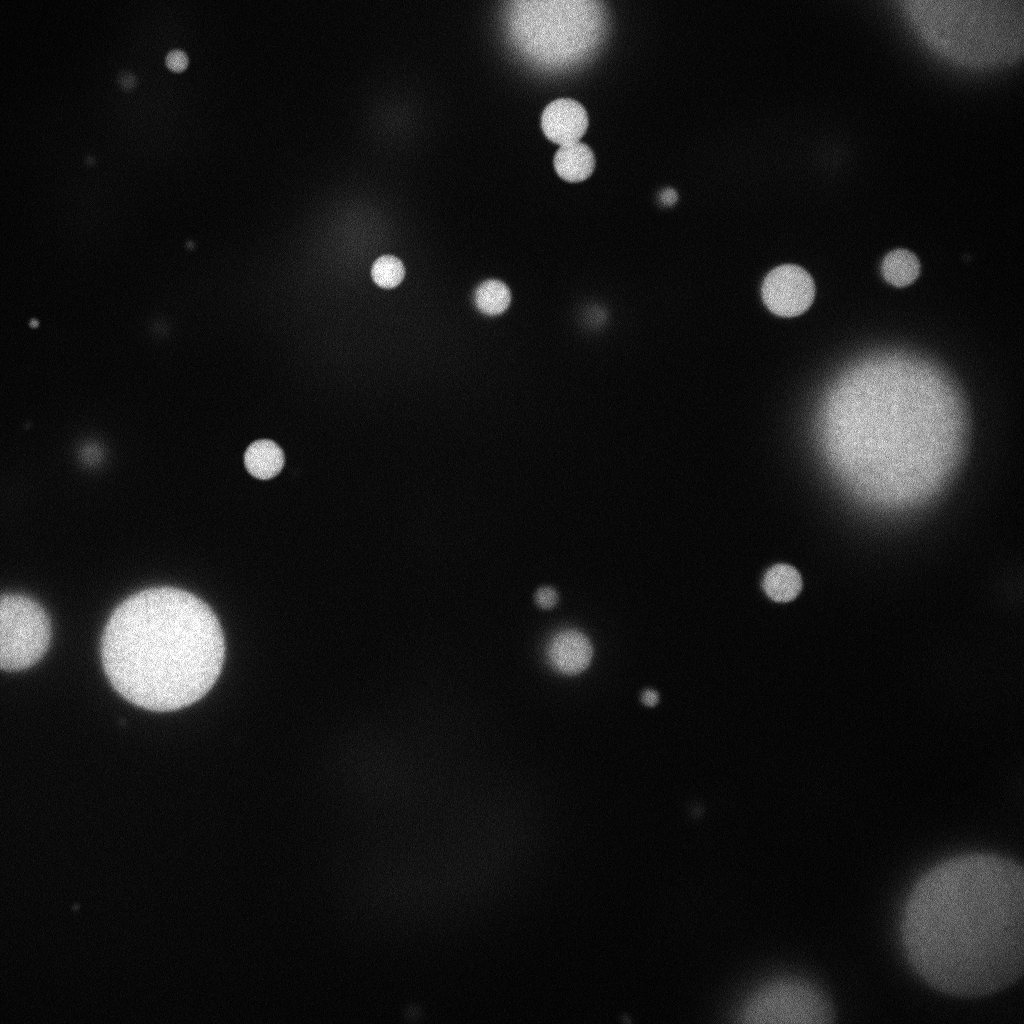

Supplement: Supplementary file 5 — Source data Fig. 1 [file 44319_2026_730_MOESM5_ESM.zip › Figure 1/1D/D1-D2-RGG.tif]

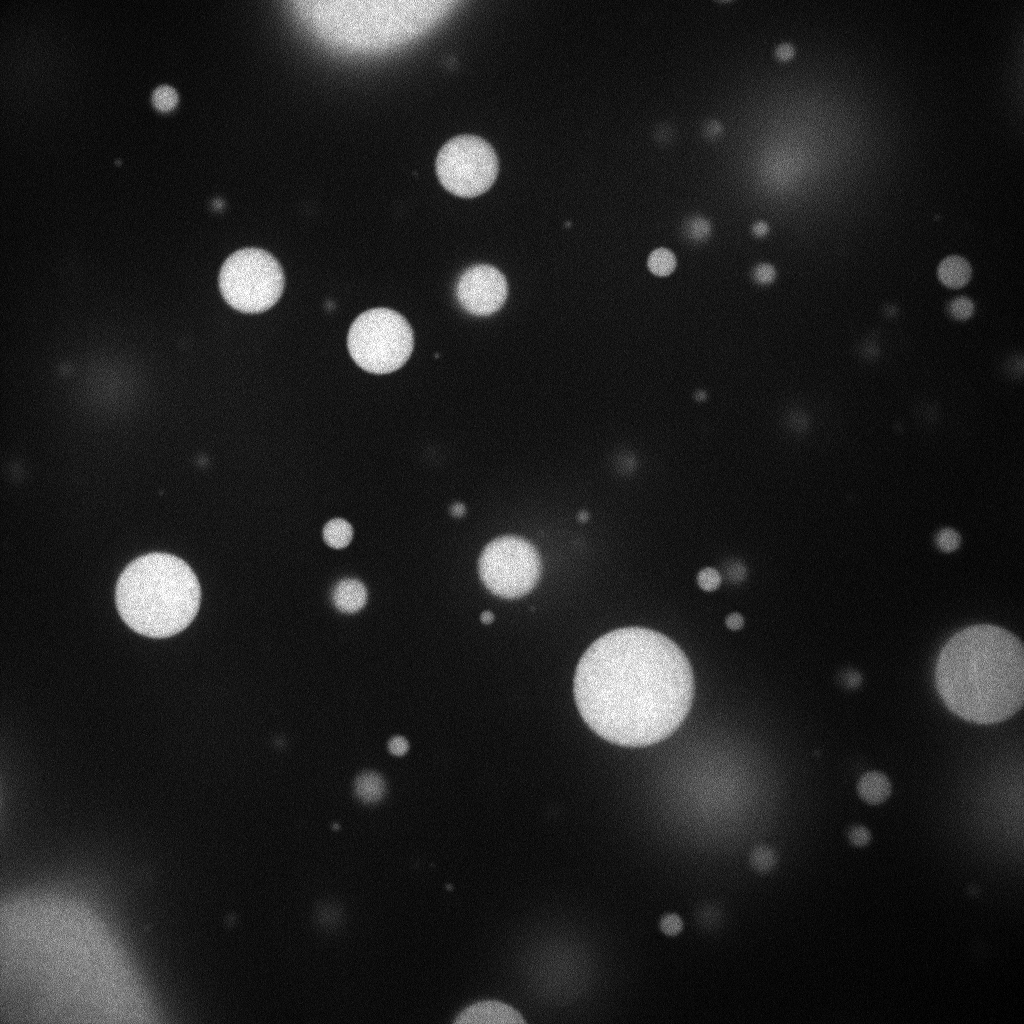

Supplement: Supplementary file 5 — Source data Fig. 1 [file 44319_2026_730_MOESM5_ESM.zip › Figure 1/1D/FL.tif]

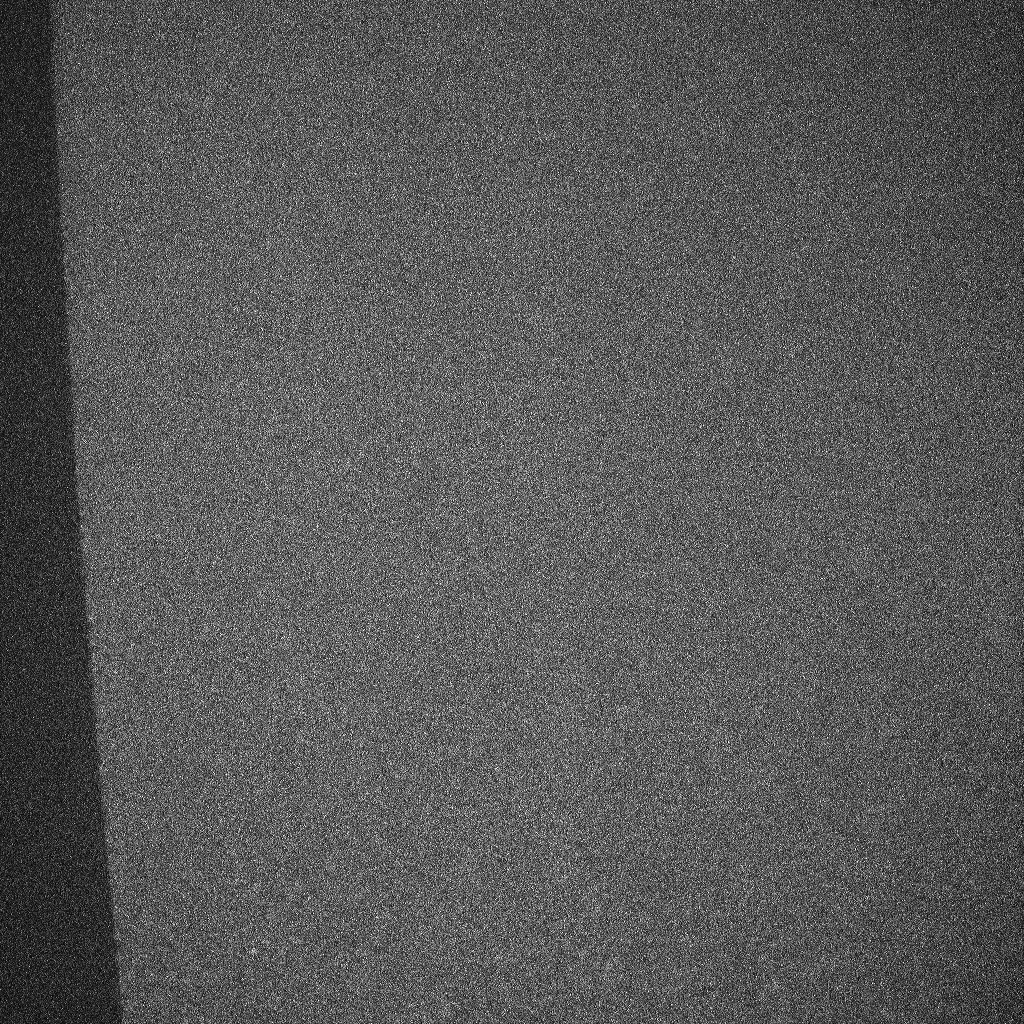

Supplement: Supplementary file 5 — Source data Fig. 1 [file 44319_2026_730_MOESM5_ESM.zip › Figure 1/1D/D2.tif]

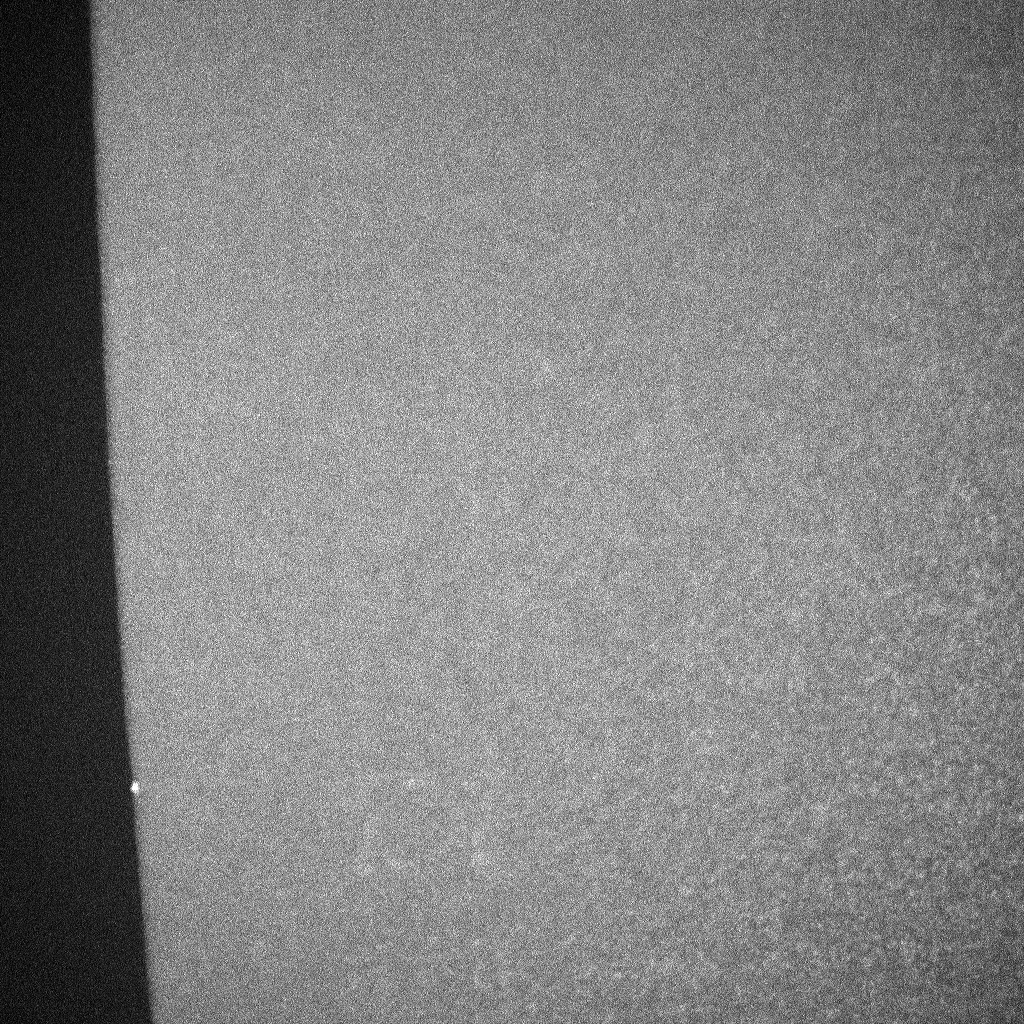

Supplement: Supplementary file 5 — Source data Fig. 1 [file 44319_2026_730_MOESM5_ESM.zip › Figure 1/1D/D1-IDR-RGG.tif]

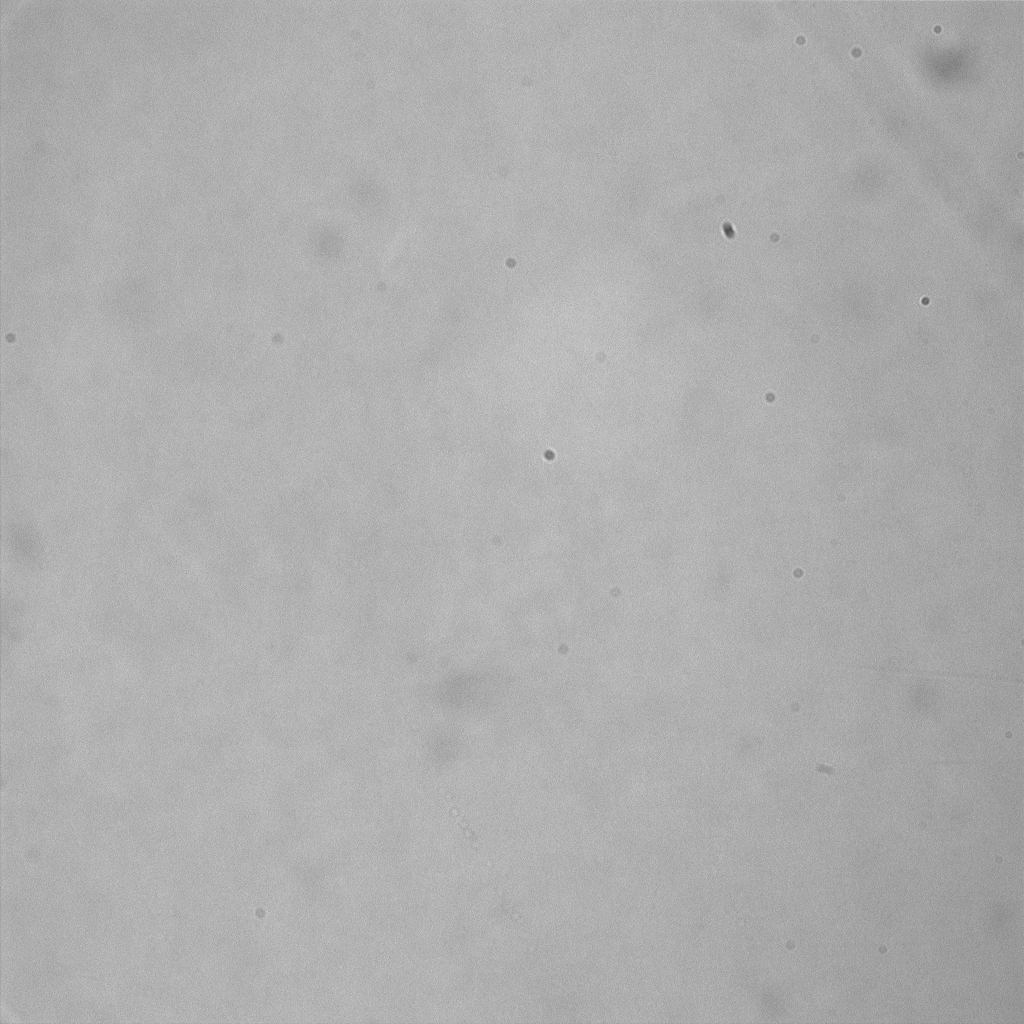

Supplement: Supplementary file 7 — Source data Fig. 3 [file 44319_2026_730_MOESM7_ESM.zip › Figure 3/3A/D1D2-IDR 5-0 - 2.tif]

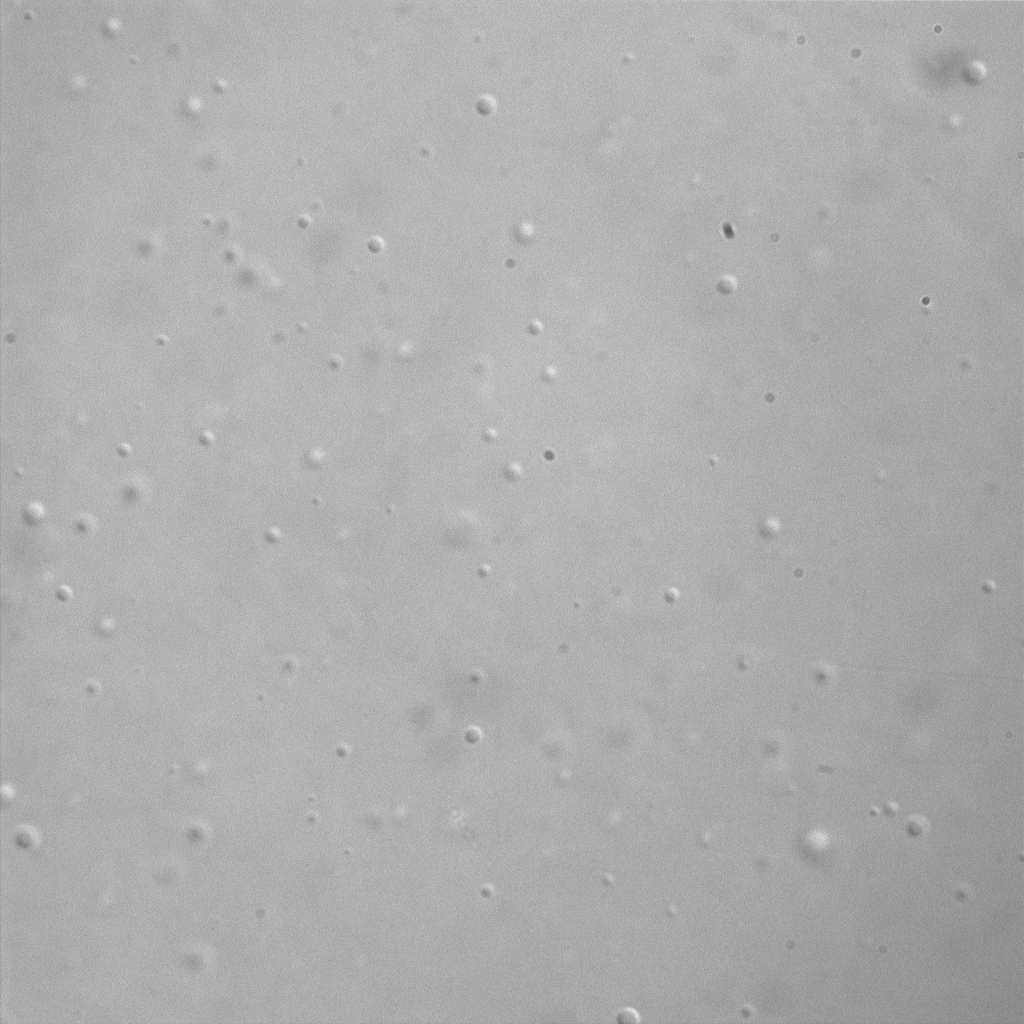

Supplement: Supplementary file 7 — Source data Fig. 3 [file 44319_2026_730_MOESM7_ESM.zip › Figure 3/3A/FL 5-0.tif]

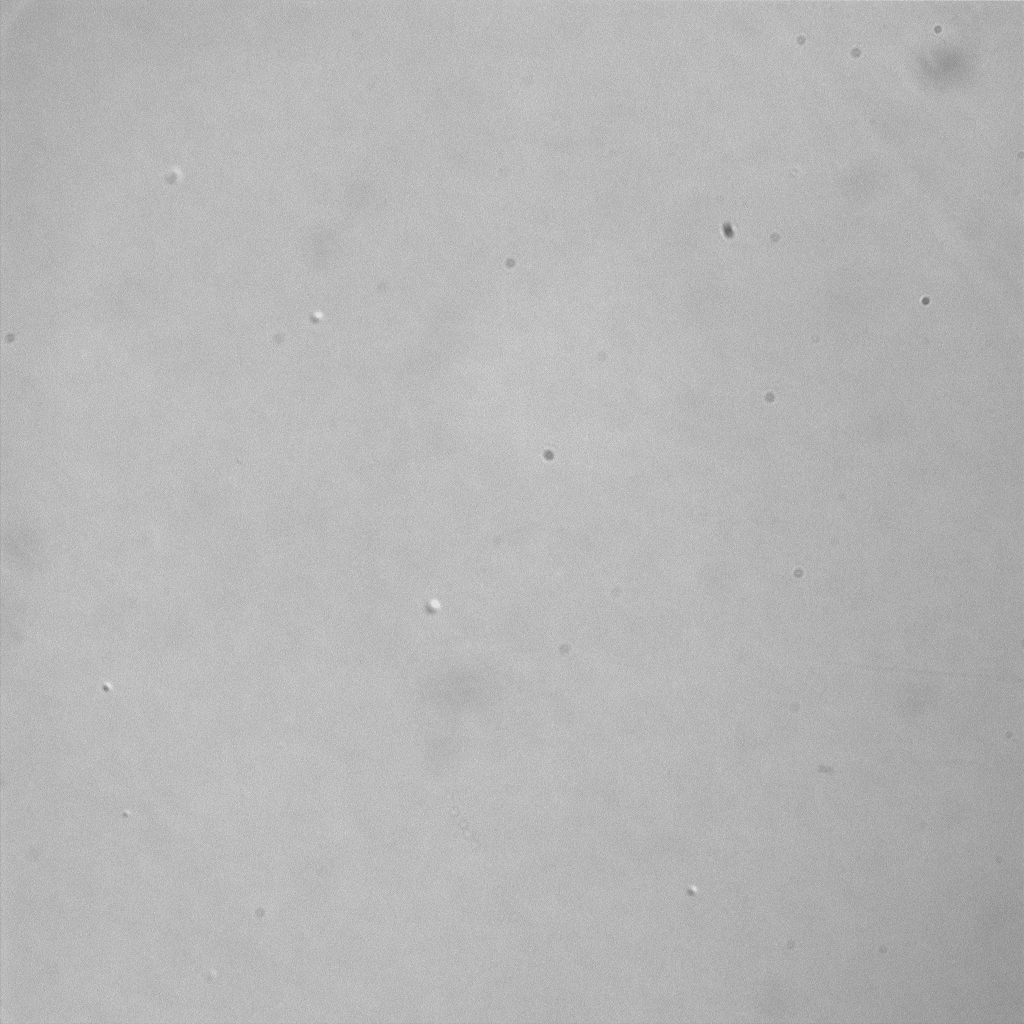

Supplement: Supplementary file 7 — Source data Fig. 3 [file 44319_2026_730_MOESM7_ESM.zip › Figure 3/3A/D1D2-RGG 1-0 - 3.tif]

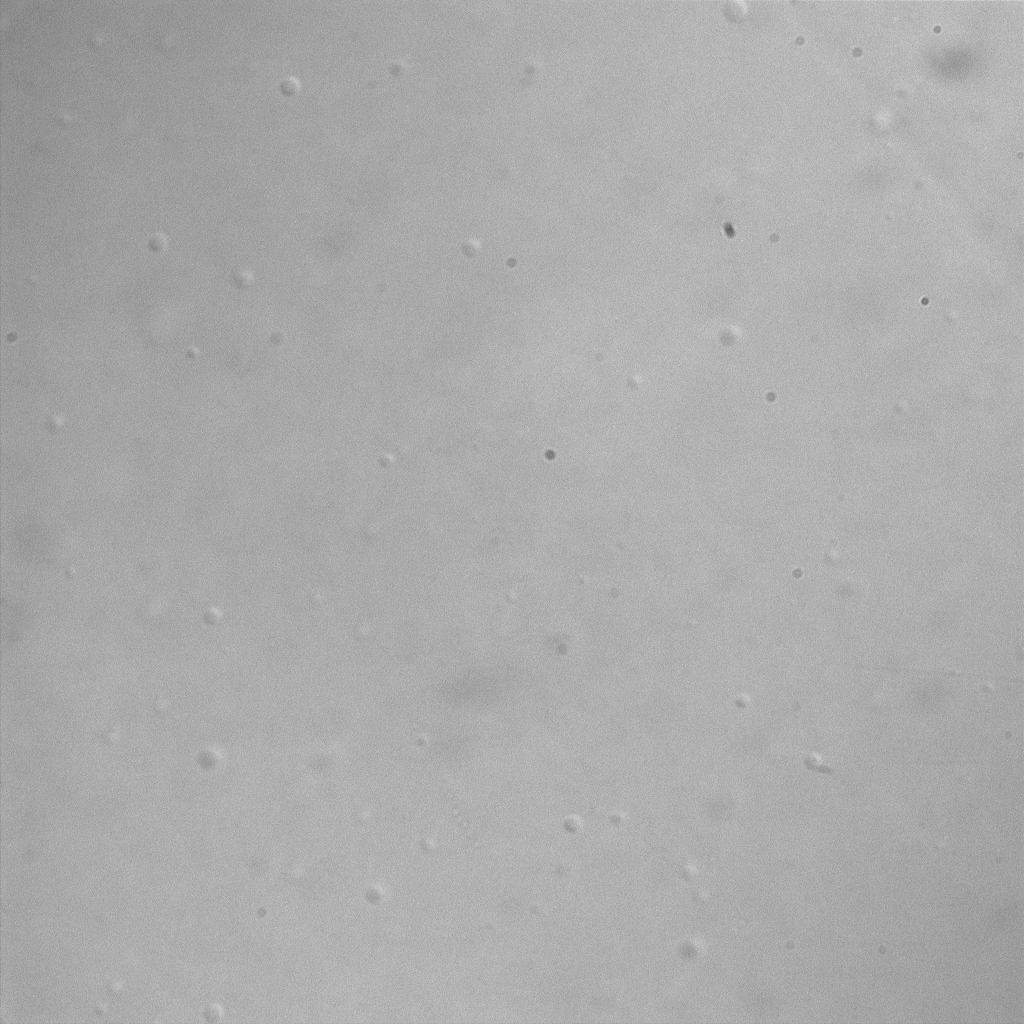

Supplement: Supplementary file 7 — Source data Fig. 3 [file 44319_2026_730_MOESM7_ESM.zip › Figure 3/3A/D1D2 5-0 - 1.tif]

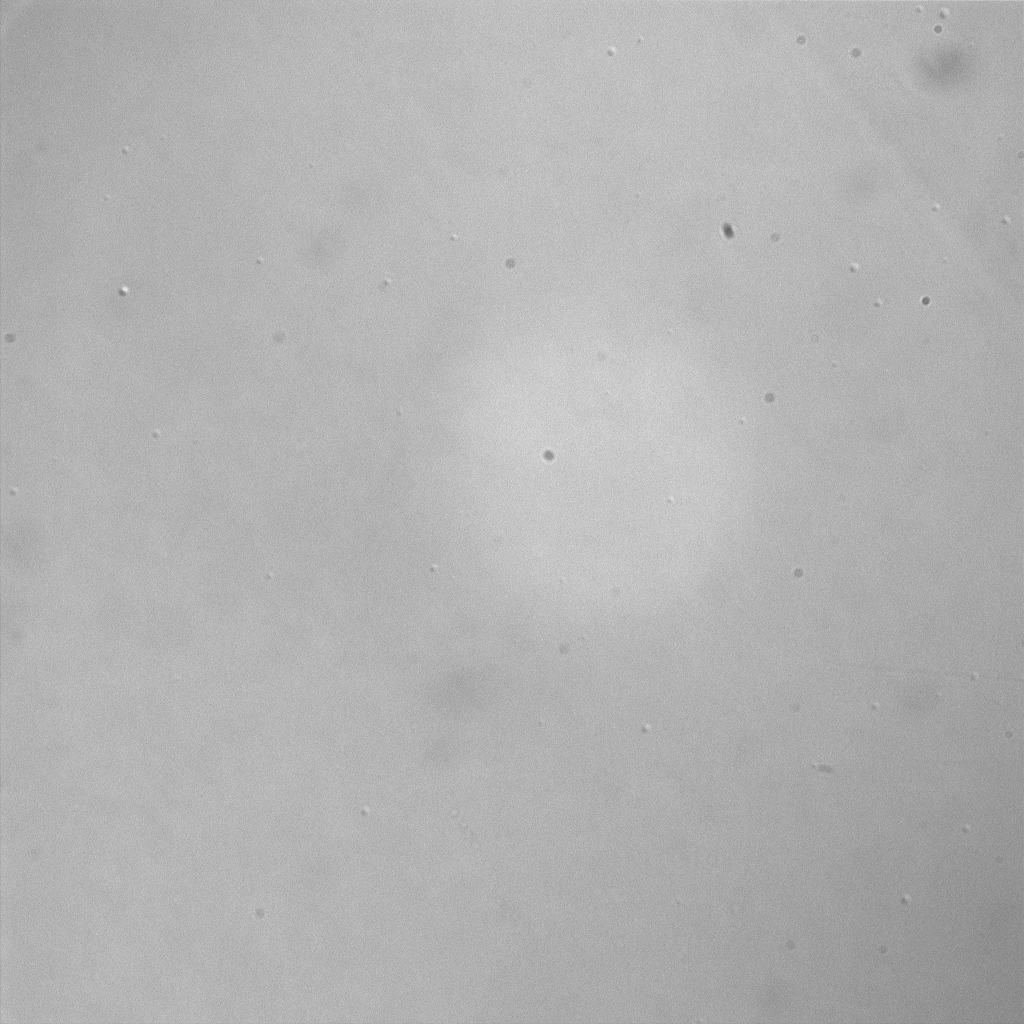

Supplement: Supplementary file 7 — Source data Fig. 3 [file 44319_2026_730_MOESM7_ESM.zip › Figure 3/3A/D1D2-3GS-RGG 1-0 - 1.tif]

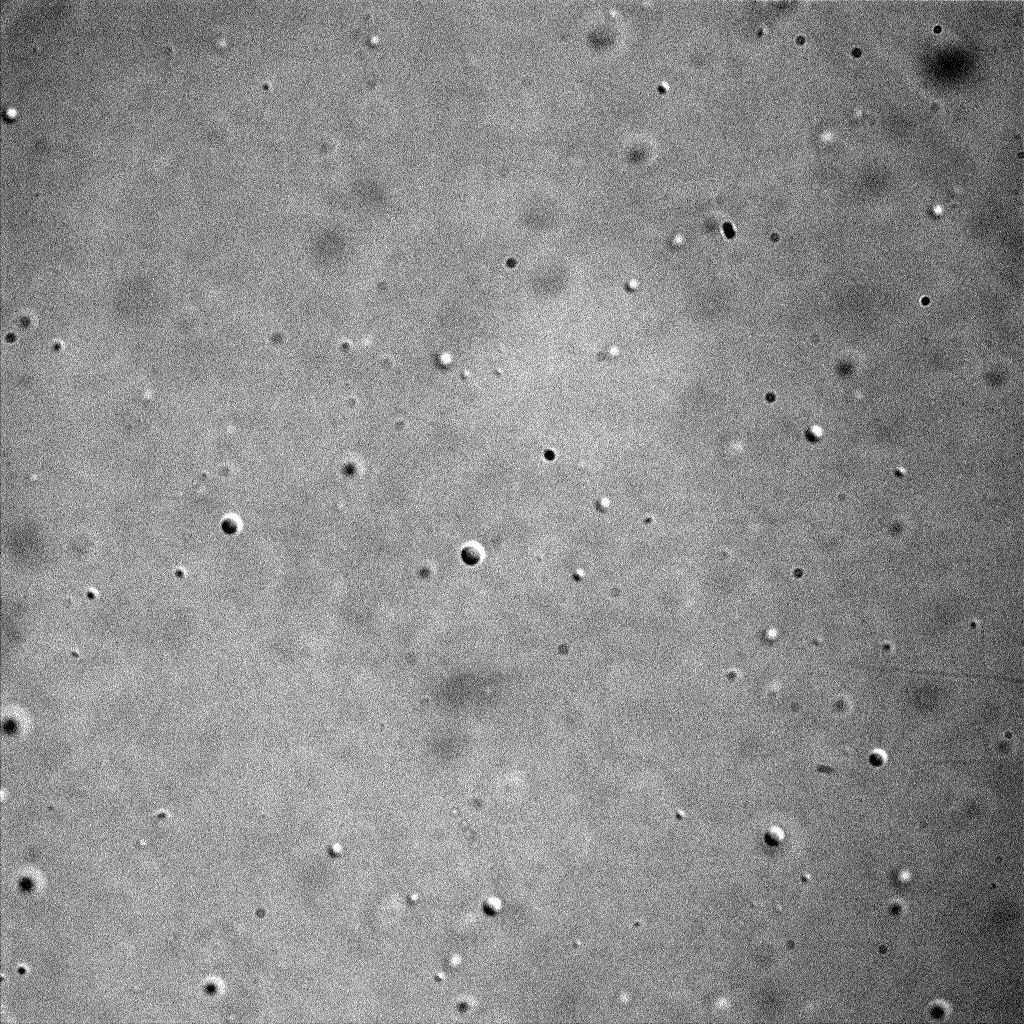

Supplement: Supplementary file 7 — Source data Fig. 3 [file 44319_2026_730_MOESM7_ESM.zip › Figure 3/3A/D1D2-3GS-RGG 5 0.tif]

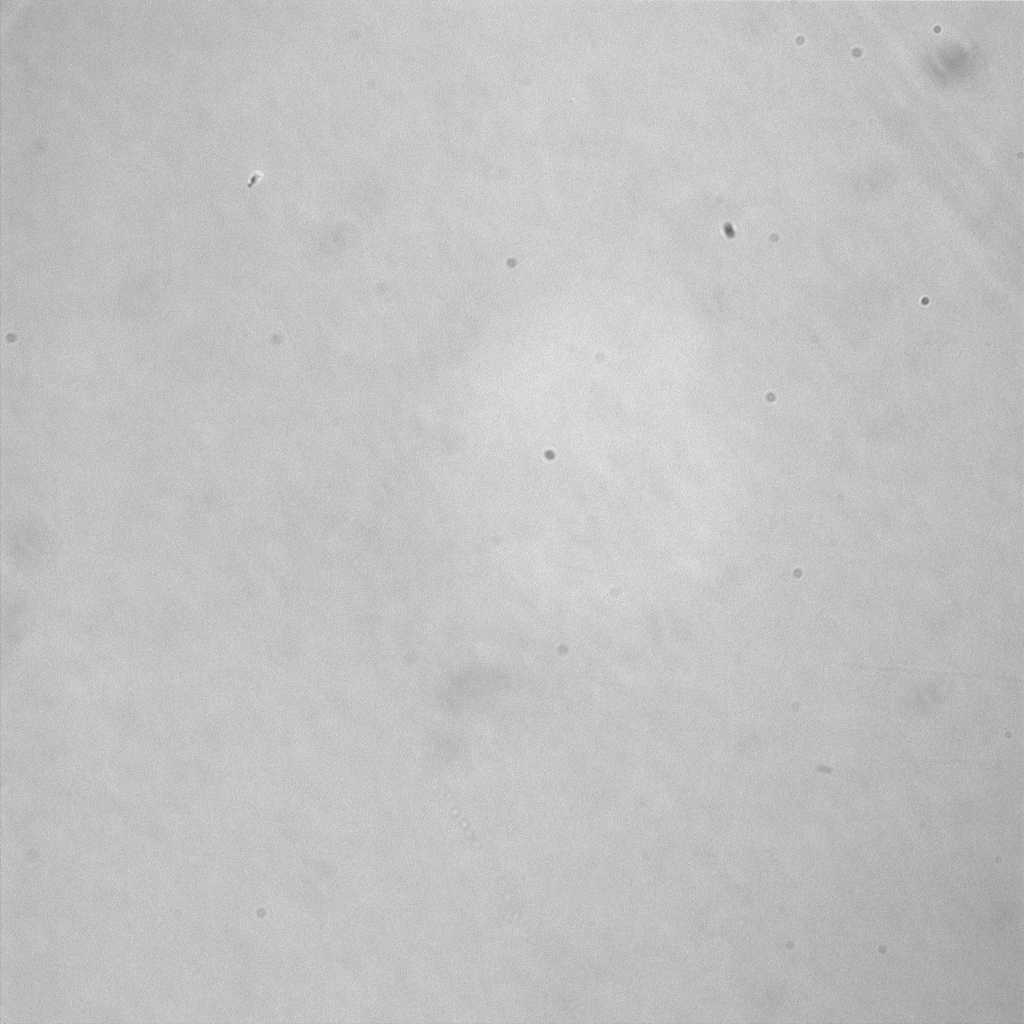

Supplement: Supplementary file 7 — Source data Fig. 3 [file 44319_2026_730_MOESM7_ESM.zip › Figure 3/3A/D1D2 1-0.tif]

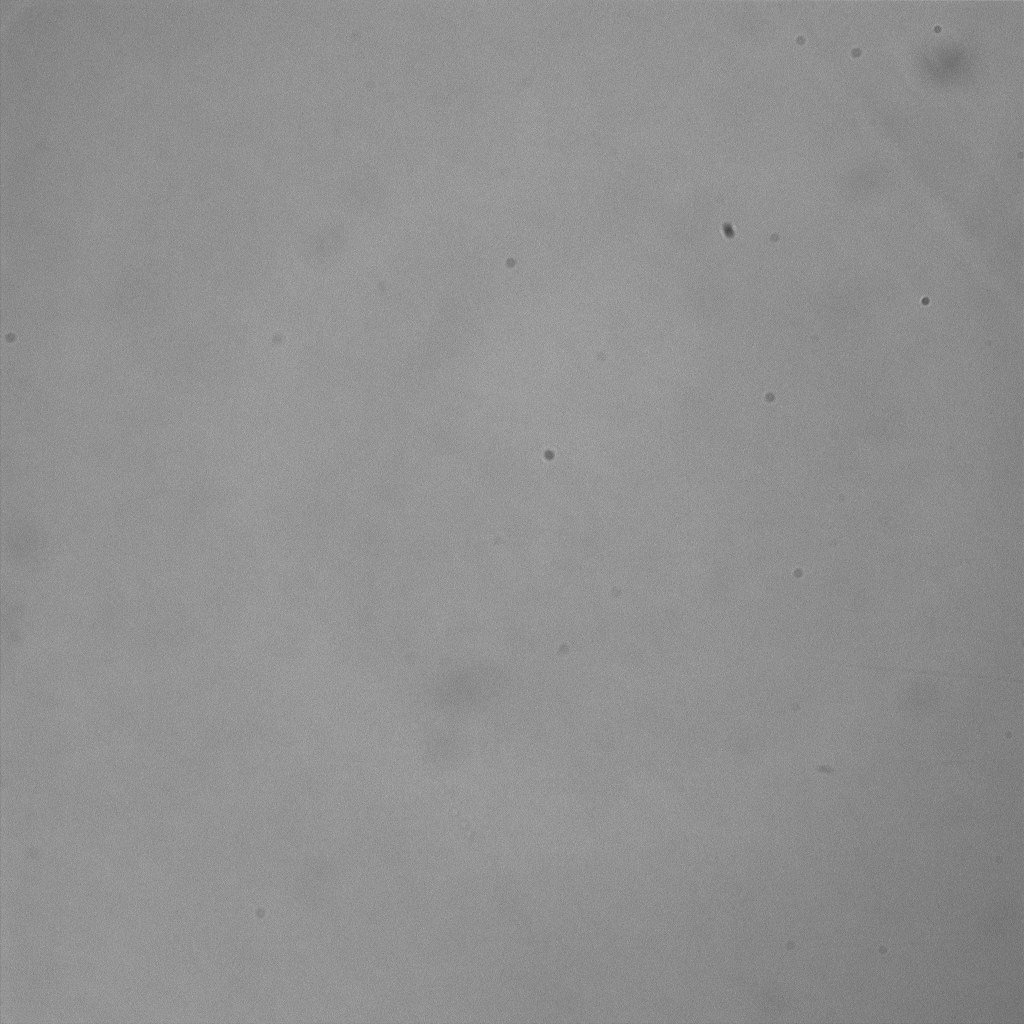

Supplement: Supplementary file 7 — Source data Fig. 3 [file 44319_2026_730_MOESM7_ESM.zip › Figure 3/3A/D1D2-IDR 1-0 - 2.tif]

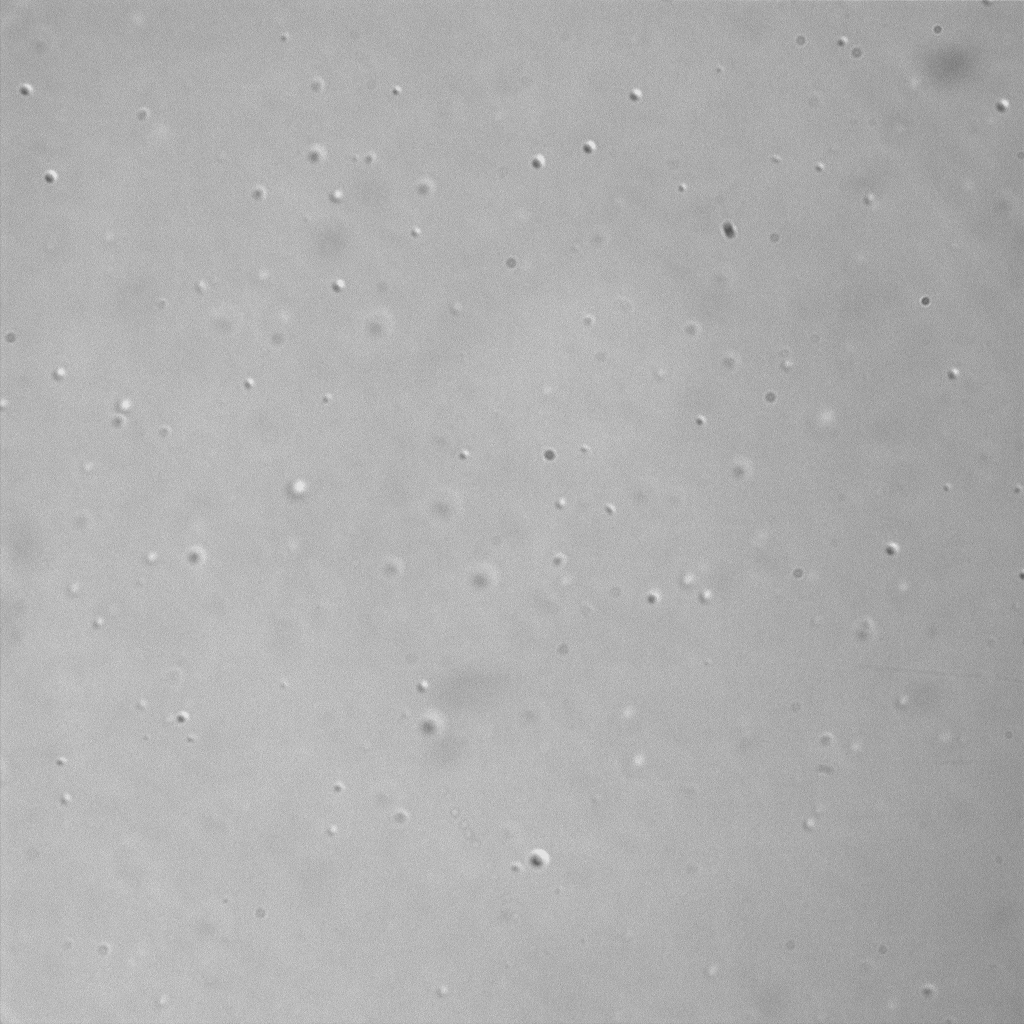

Supplement: Supplementary file 7 — Source data Fig. 3 [file 44319_2026_730_MOESM7_ESM.zip › Figure 3/3A/D1D2-RGG 5-0 - 1.tif]

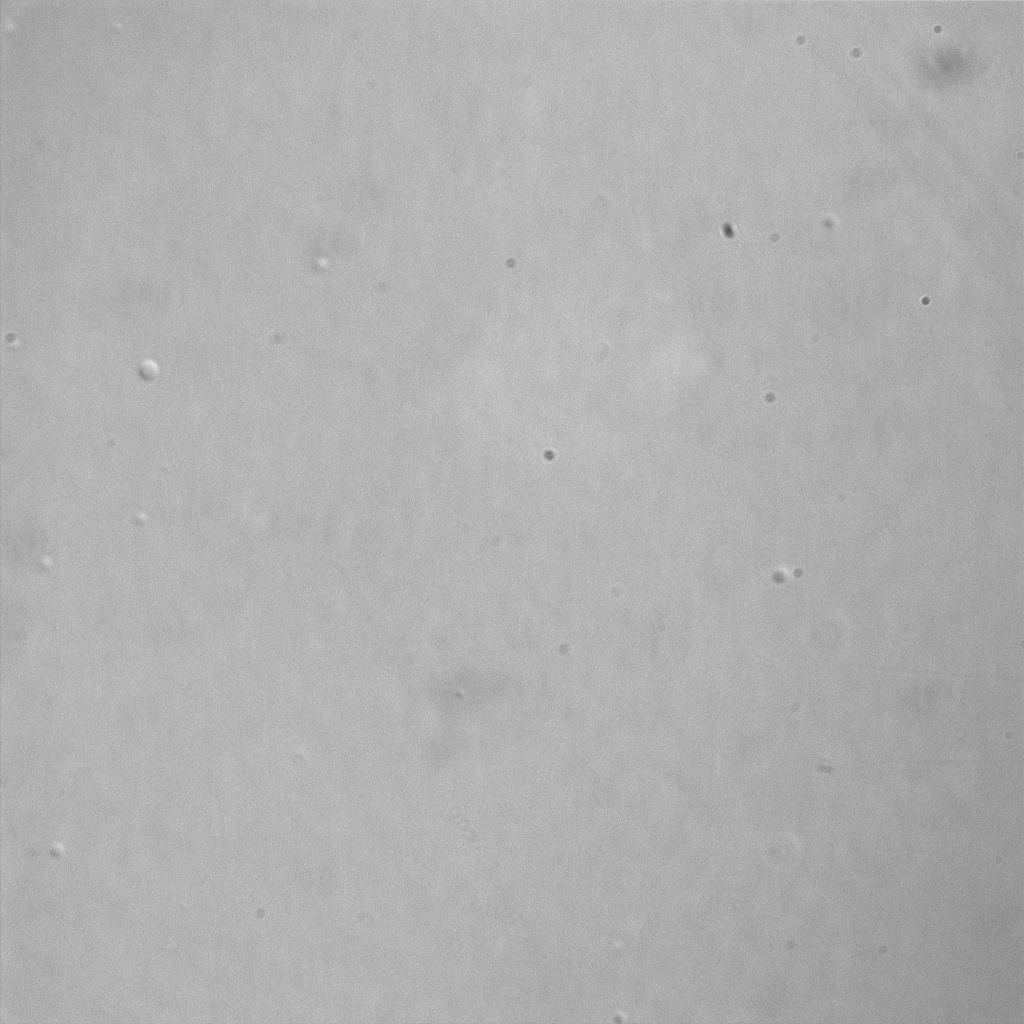

Supplement: Supplementary file 7 — Source data Fig. 3 [file 44319_2026_730_MOESM7_ESM.zip › Figure 3/3A/FL 1-0 2.tif]

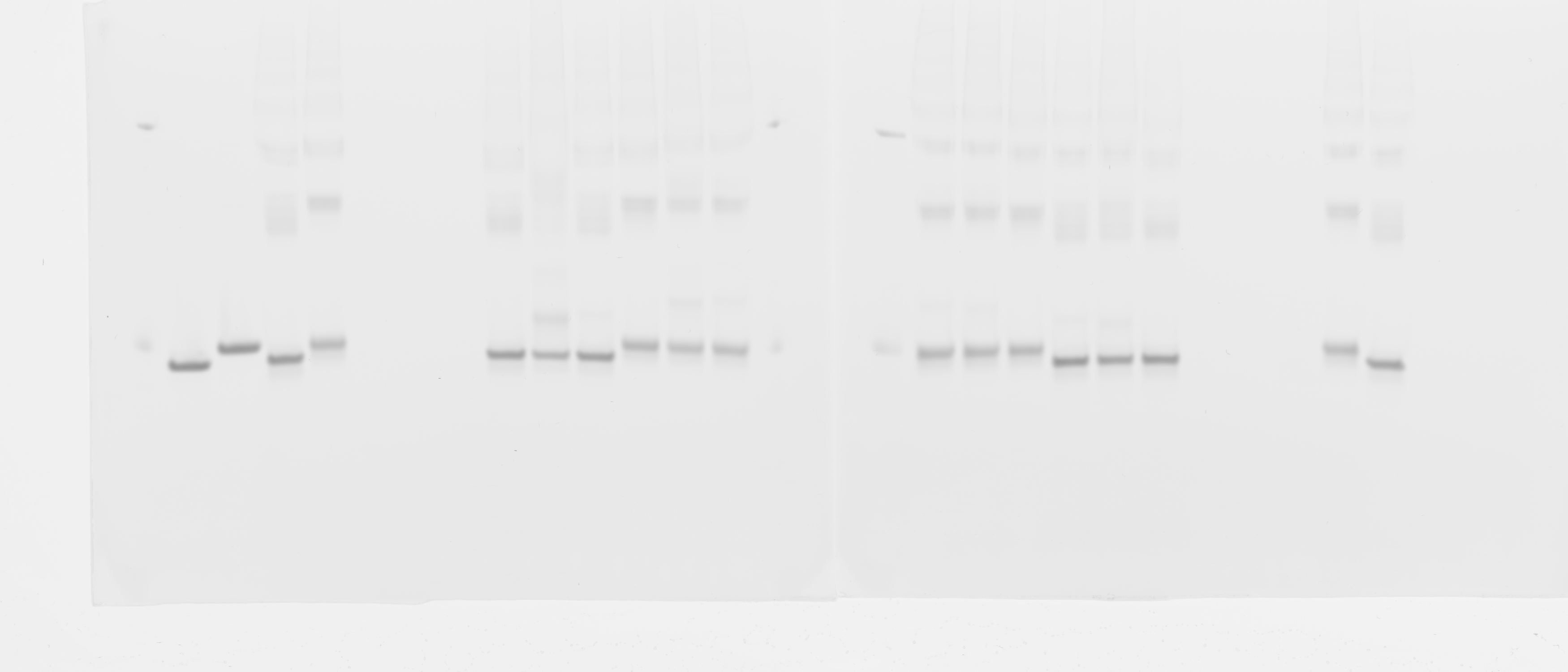

Supplement: Supplementary file 8 — Source data Fig. 5 [file 44319_2026_730_MOESM8_ESM.zip › Figure 5/5A/DL488, FA crosslinkin PMT600 20250129-163821-[Cy2].gel]

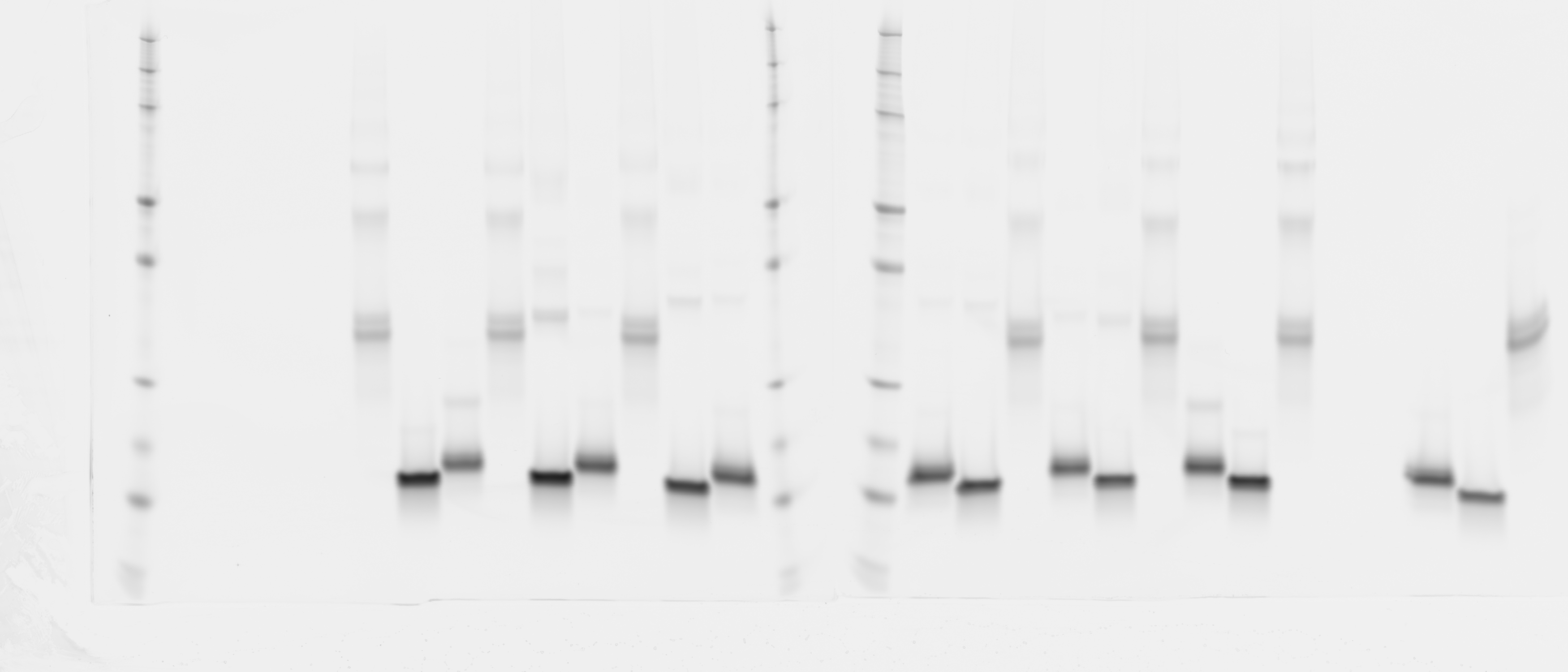

Supplement: Supplementary file 8 — Source data Fig. 5 [file 44319_2026_730_MOESM8_ESM.zip › Figure 5/5A/AF647, FA crosslinking PMT600 20250129-163821-[Cy5].gel]

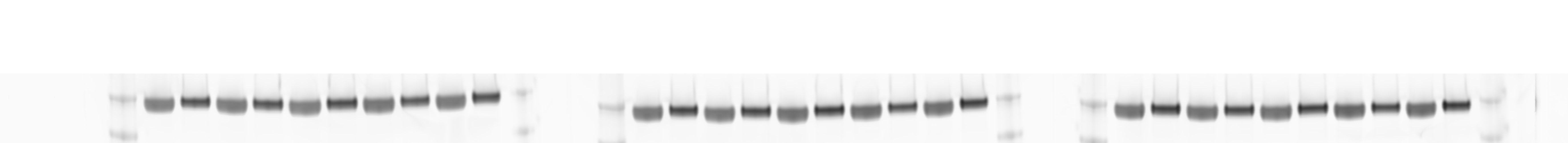

Supplement: Supplementary file 8 — Source data Fig. 5 [file 44319_2026_730_MOESM8_ESM.zip › Figure 5/5D/In-trans pelleting assay PMT450 20241017-191736-[Cy5].gel]

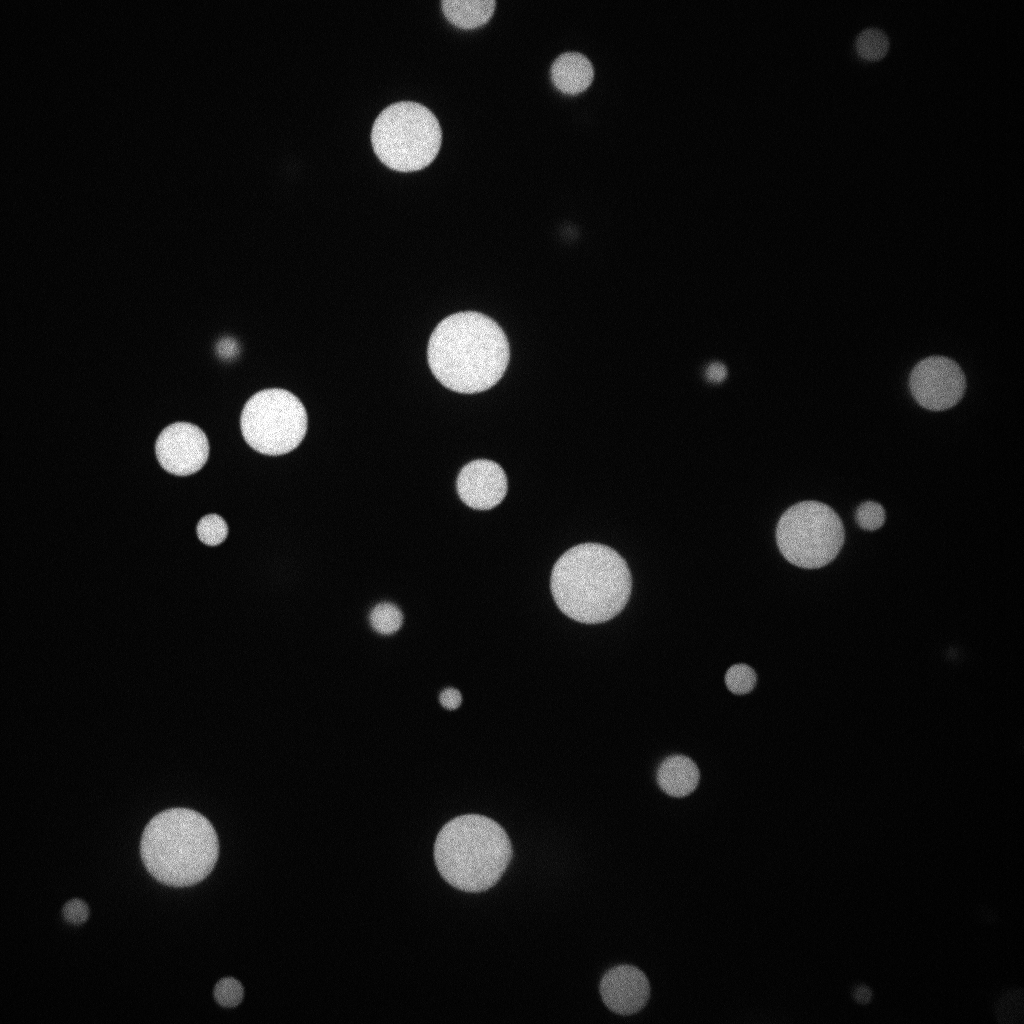

Supplement: Supplementary file 8 — Source data Fig. 5 [file 44319_2026_730_MOESM8_ESM.zip › Figure 5/5B-C/5B/RGG.tif]

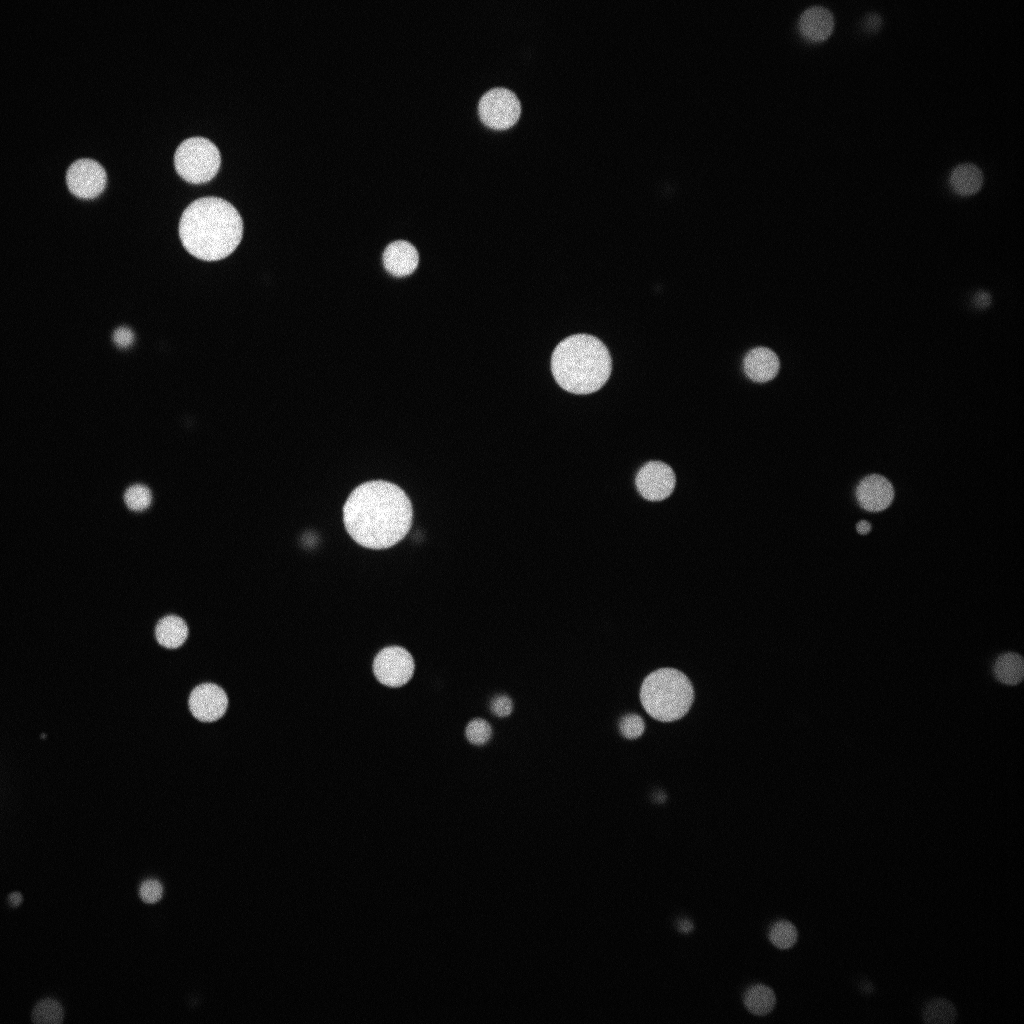

Supplement: Supplementary file 8 — Source data Fig. 5 [file 44319_2026_730_MOESM8_ESM.zip › Figure 5/5B-C/5B/FreeDye.tif]

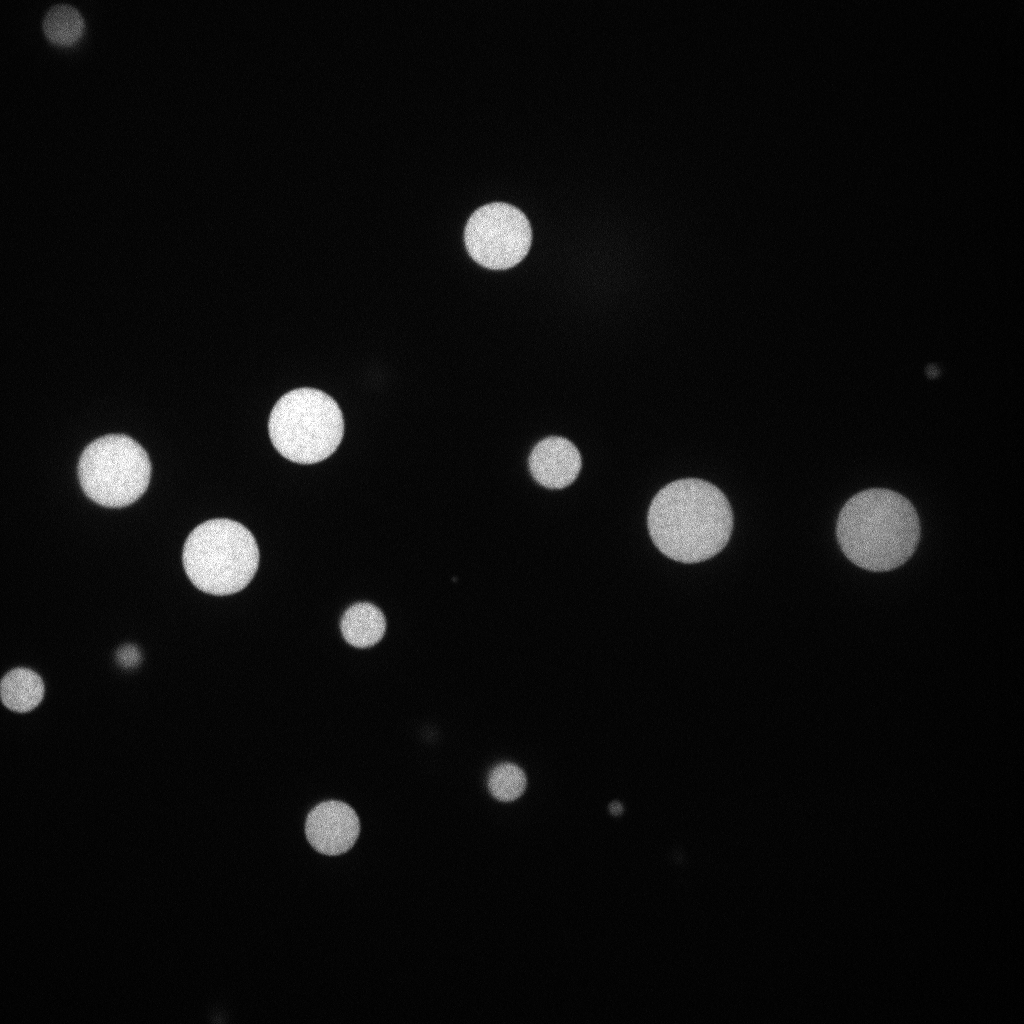

Supplement: Supplementary file 8 — Source data Fig. 5 [file 44319_2026_730_MOESM8_ESM.zip › Figure 5/5B-C/5B/KGG.tif]

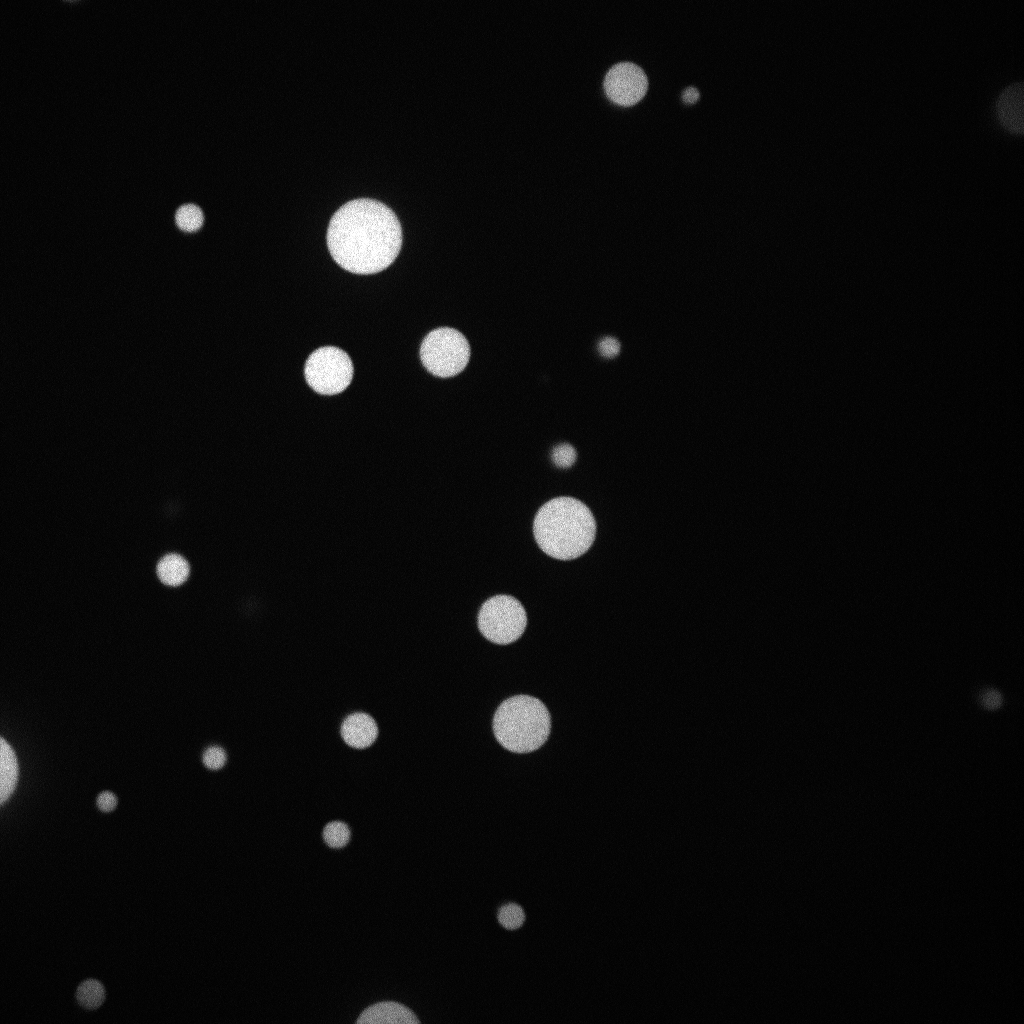

Supplement: Supplementary file 8 — Source data Fig. 5 [file 44319_2026_730_MOESM8_ESM.zip › Figure 5/5B-C/5B/IDR.tif]

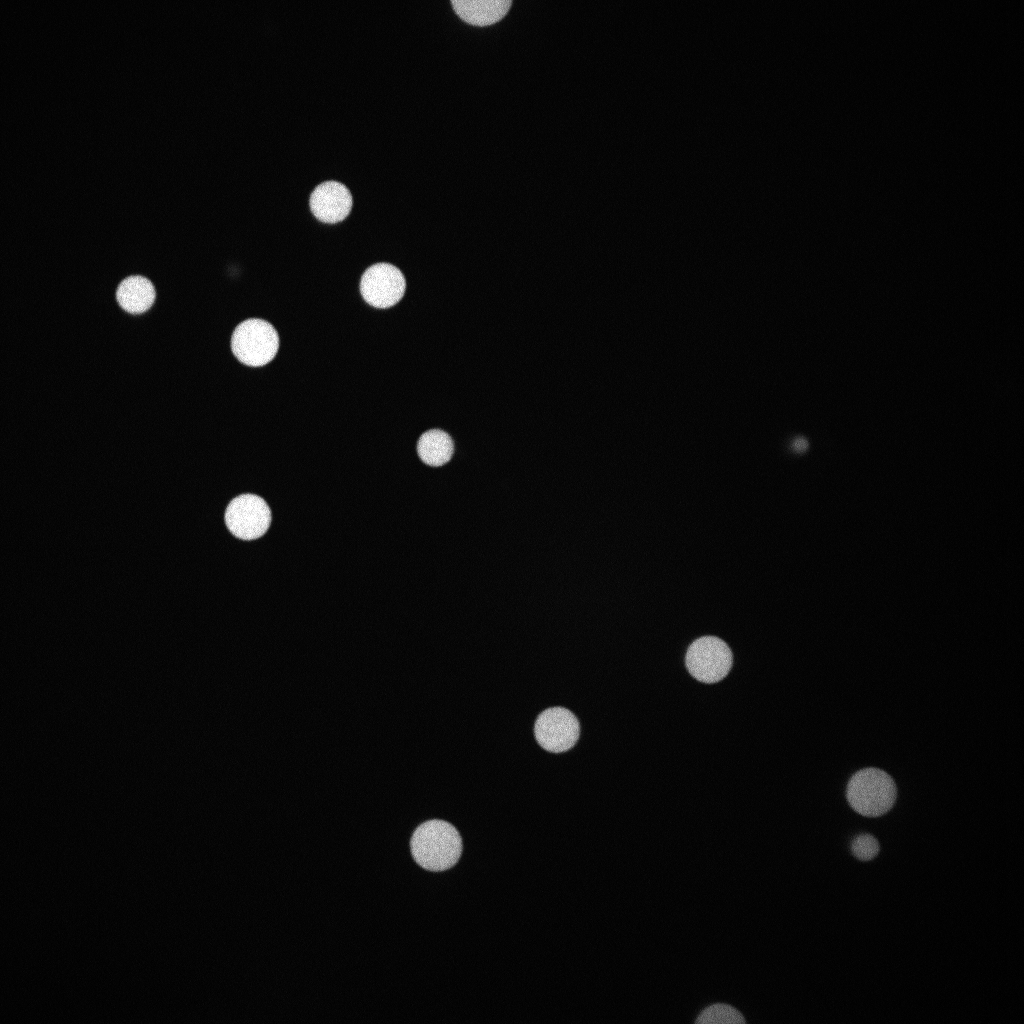

Supplement: Supplementary file 9 — Source data Fig. 6 [file 44319_2026_730_MOESM9_ESM.zip › Figure 6/6B/300 ng RNA per ┬╡l.tif]

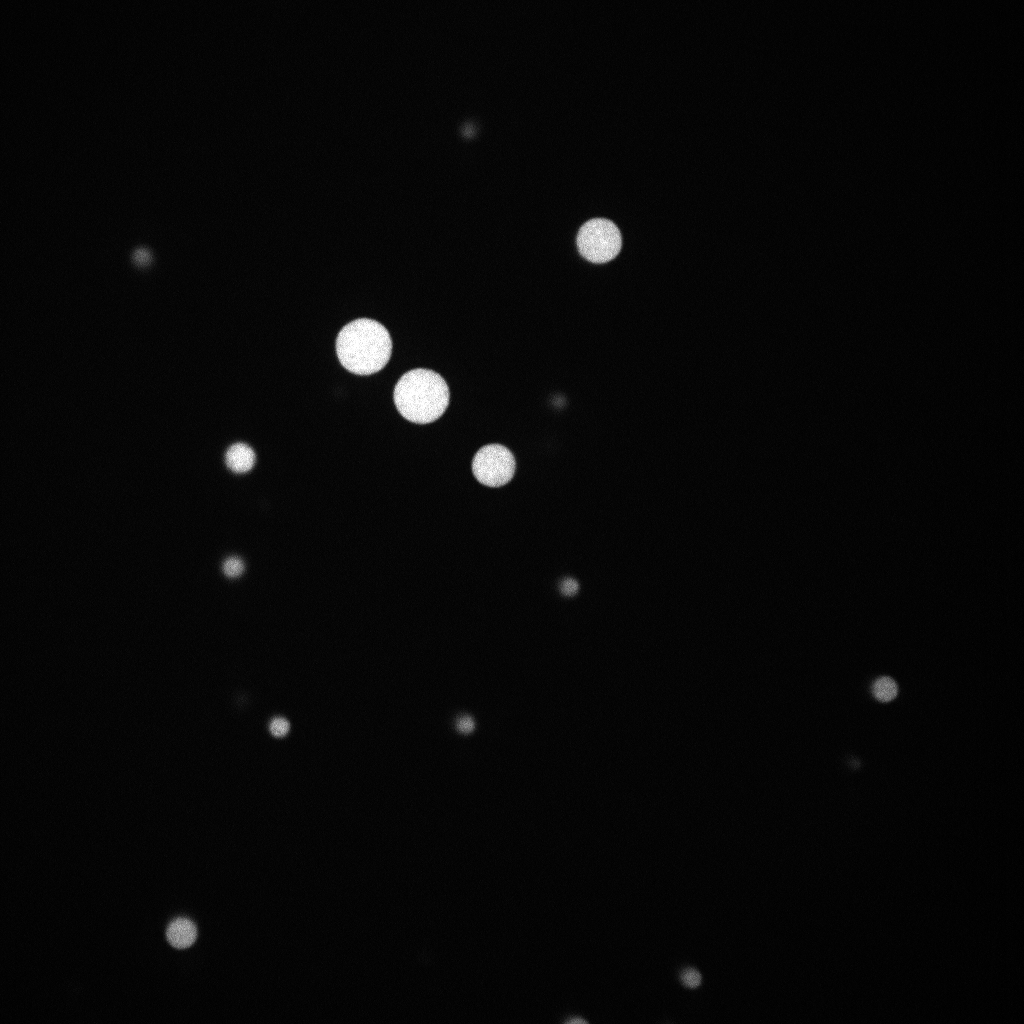

Supplement: Supplementary file 9 — Source data Fig. 6 [file 44319_2026_730_MOESM9_ESM.zip › Figure 6/6B/0 ng RNA per ┬╡l.tif]

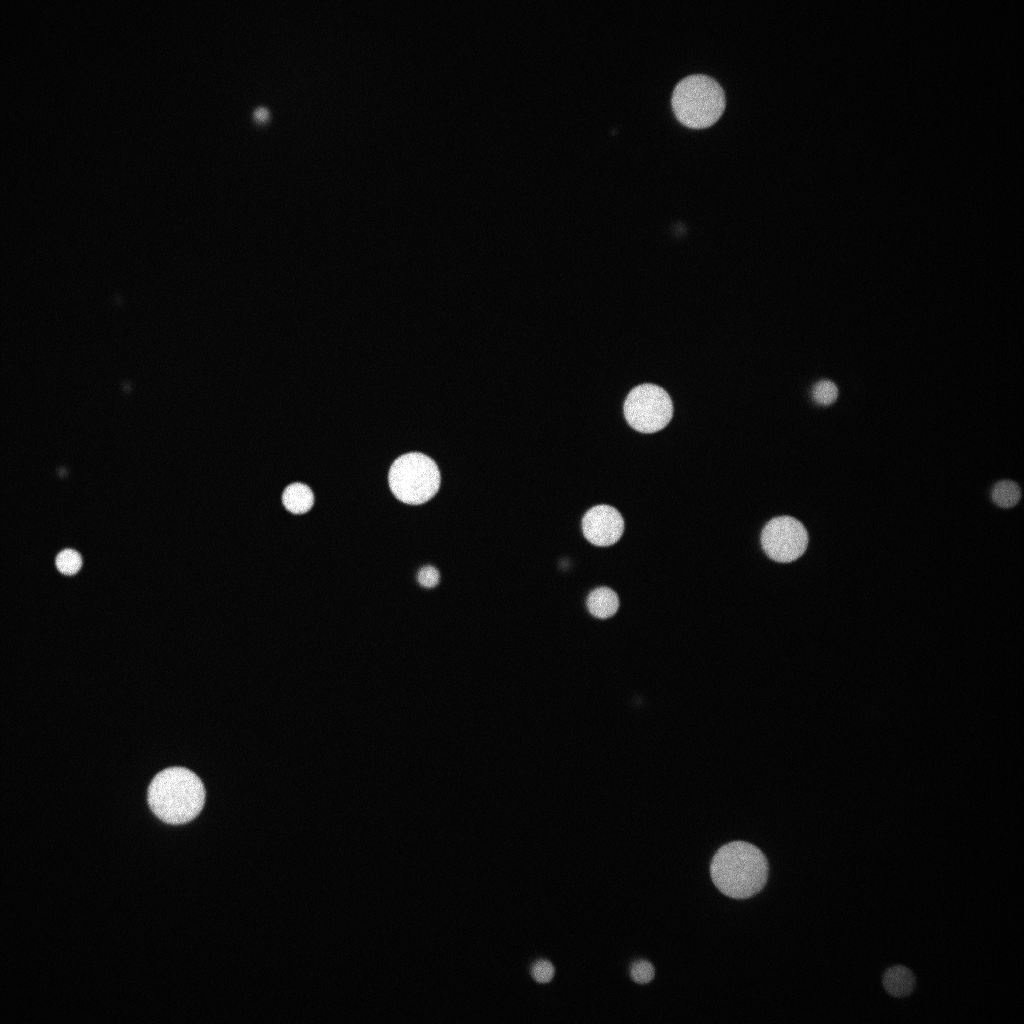

Supplement: Supplementary file 9 — Source data Fig. 6 [file 44319_2026_730_MOESM9_ESM.zip › Figure 6/6B/500 ng RNA per ┬╡l.tif]
