# Supplementary material for: Phase separation of PGL-3 driven by structured domains that oligomerize and interact with RGG motifs
Source: EMBO Rep. 2026 Mar 20;27(8):2061–87. doi: 10.1038/s44319-026-00730-7 (PMC13121748; doi:10.1038/s44319-026-00730-7)
Supplement: Supplementary file 10 — Expanded View Figures [file 44319_2026_730_MOESM10_ESM.pdf]

Expanded View Figures

Figure EV1. The IDR-RGG region does not undergo phase separation.

(**A**) Protein gel stained with Coomassie Brilliant Blue G250 showing the recombinant PGL-3 derivatives used in the condensation assays shown in Fig. 1C, D. About 1 µg protein per well was loaded. (**B**) Table listing the region and the molecular weight for each PGL-3 constructs used in this study. (**C**) DIC micrographs of unlabeled PGL-3 and its derivatives of indicated protein concentrations at 67.5 or 125 mM NaCl. Background pattern visible in all panels (and also in DIC panels in (**D**, **E**)) originates from microscope internal parts. Scale bar = 20 µm. (**D**) DIC micrographs of unlabeled PGL-3 IDR-RGG at 101.6 µM PGL-3(IDR-RGG), 125 mM NaCl and varying concentrations of *nos-2* mRNA. Scale bar = 20 µm. (**E**) DIC micrographs of unlabeled wild-type and D1 dimerization mutant of full-length PGL-3 tested for condensation at the indicated protein and salt concentrations. Note the robust condensation of wild-type PGL-3, which is not observed for the mutant. PGL-3(R123E, K126E, K129E) is full-length PGL-3 with mutations in the D1 domain, previously shown to interfere with dimerization in PGL-1 (Aoki et al, 2021). Scale bar = 20 µm.

© The Author(s)

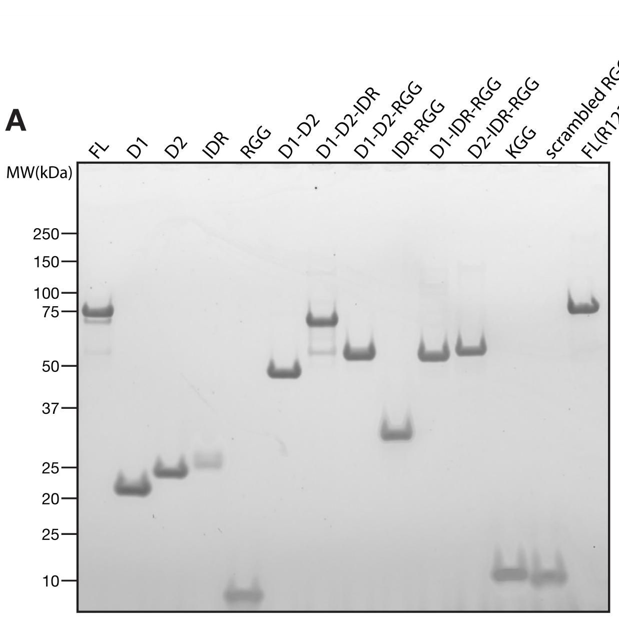

B

Construct	Residues	MW (kDa)
FL	1-693	74.8
D1	1-212	23.5
D2	205-452	28.0
IDR	448-622	18.1
RGG	623-693	6.76
D1-D2	1-447	50.0
D1-D2-IDR	1-622	68.1
D1-D2-RGG	1-447::623-693	56.78
IDR-RGG	448-693	24.8
D1-IDR-RGG	1-212::448-693	48.3
D2-IDR-RGG	205-693	51.9
KGG	623-693(all R to K)	6.39
scrambled RGG	623-693(scrambled)	6.76

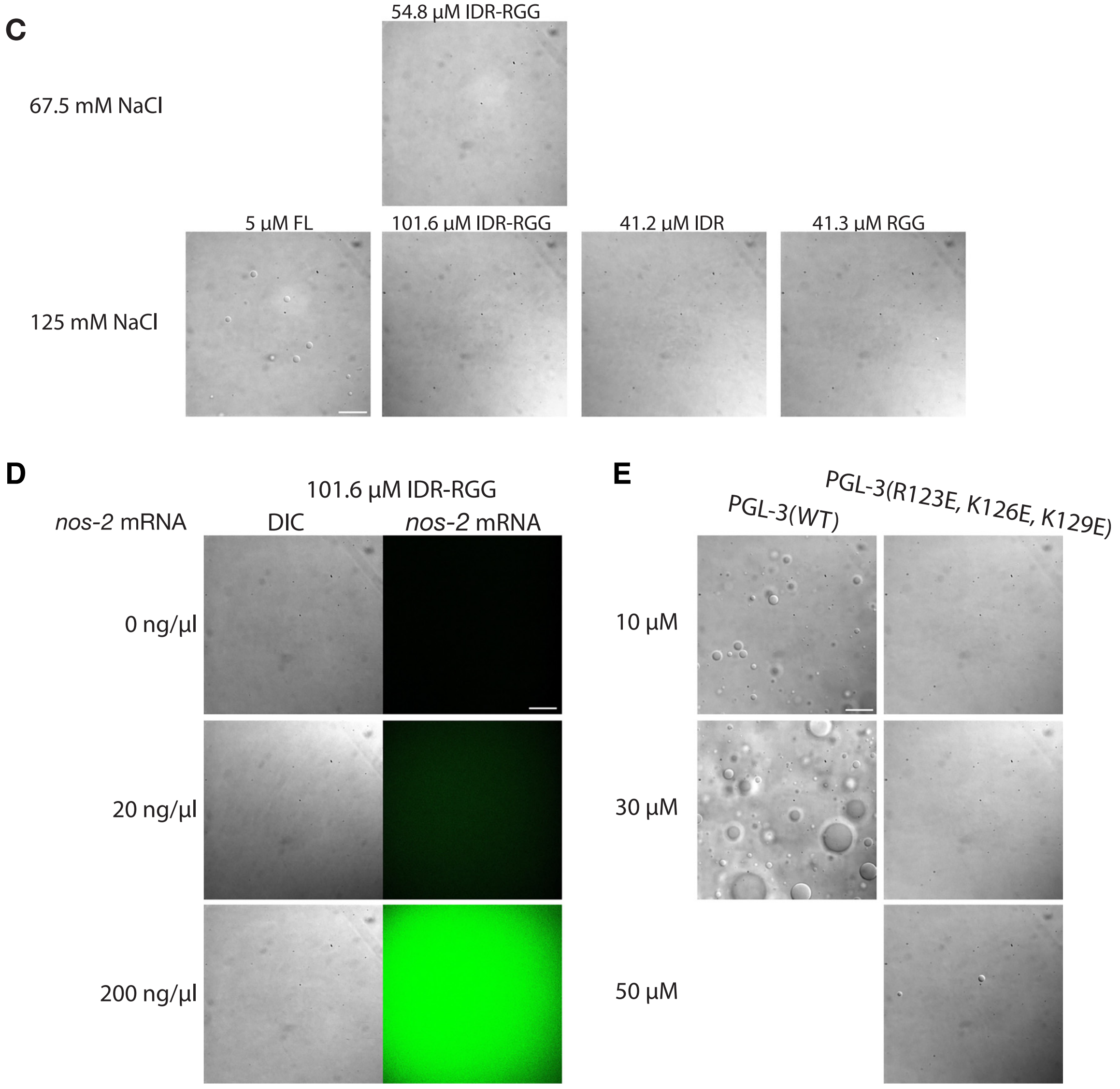

 © The Author(s)

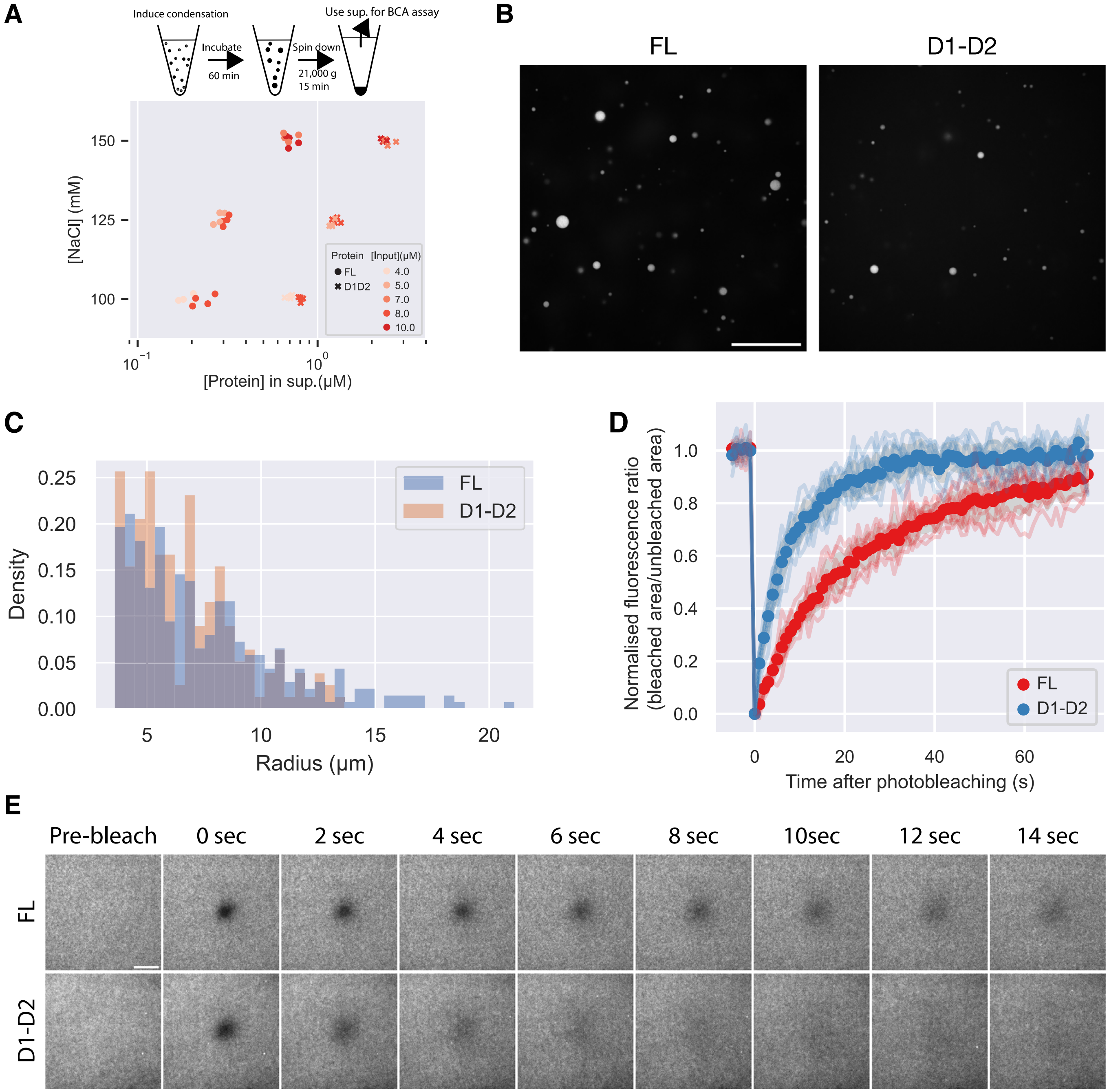

Figure EV2. Further comparison of PGL-3 FL and D1-D2 condensates.

(**A**) Top: Schematic describing the procedure of c_{sat} estimation by c_{dil} measurement. Bottom: Plot showing c_{dil} of full-length PGL-3 and D1-D2 at varying protein/salt concentrations permissive for condensation. (**B**) Representative photomicrograph showing condensates of full-length PGL-3 or D1-D2 at 5 μM protein, 125 mM NaCl. Scale bar = 50 μm. (**C**) Density histogram showing size distribution of PGL-3 condensates. *N* = 314 for full-length PGL-3, *N* = 178 for D1-D2. (**D**) Plot showing FRAP of full-length and D1-D2 condensates. Thin lines show individual traces from single condensates, dots show means, and the gray areas show standard deviation. *N* > 9. (**E**) Representative time-lapse photomicrographs showing fluorescence recovery after photobleaching (FRAP) of a 1 μm diameter circular area within a condensate. Note the faster recovery in the D1-D2 condensate. Scale bar = 5 μm.

© The Author(s)

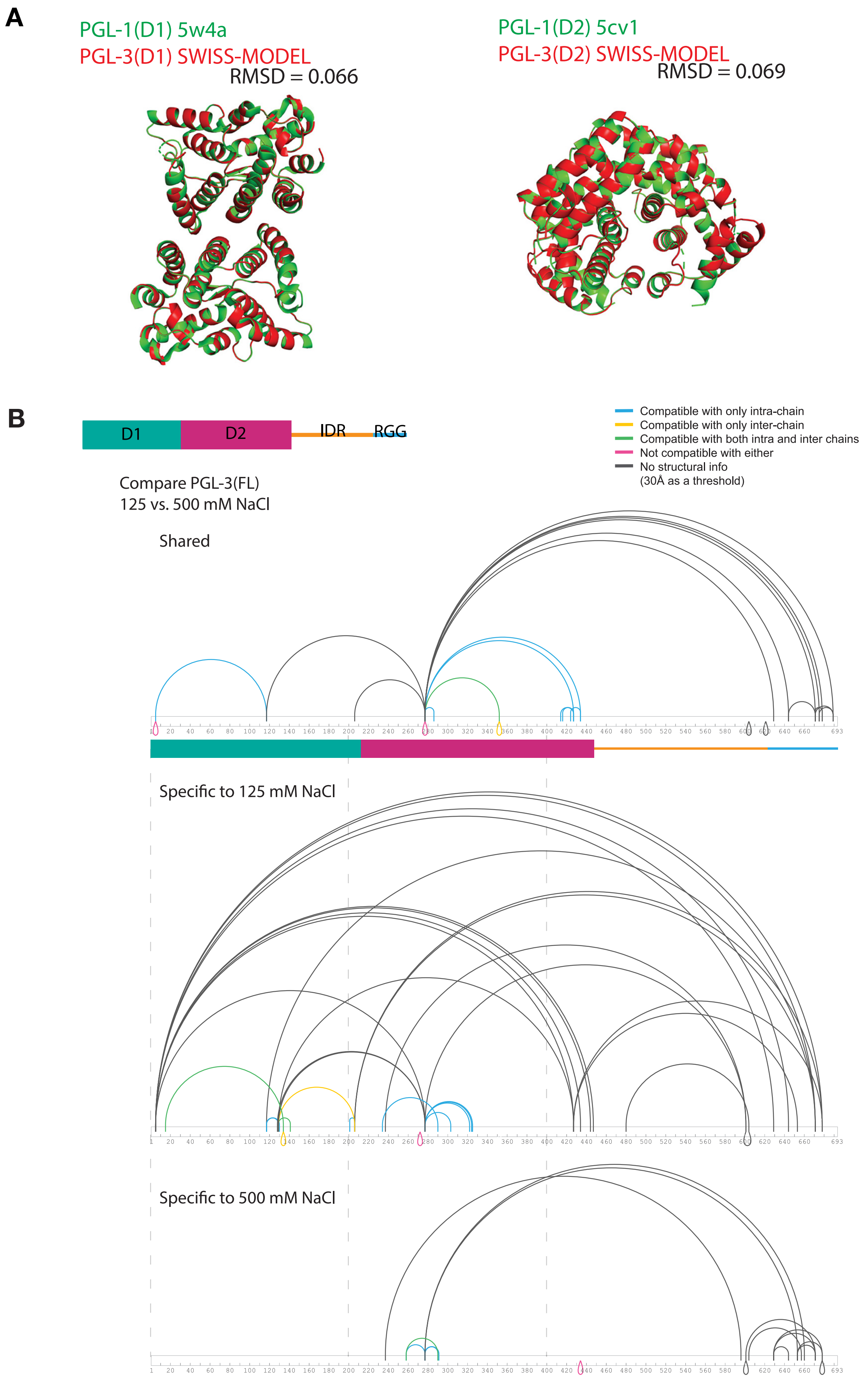
A
PGL-1(D1) 5w4a
PGL-3(D1) SWISS-MODEL
RMSD = 0.066
PGL-1(D2) 5cv1
PGL-3(D2) SWISS-MODEL
RMSD = 0.069
B
D1
D2
IDR
RGG
Compatible with only intra-chain
Compatible with only inter-chain
Compatible with both intra and inter chains
Not compatible with either
No structural info
(30Å as a threshold)
Compare PGL-3(FL)
125 vs. 500 mM NaCl
Shared
Specific to 125 mM NaCl
Specific to 500 mM NaCl

 © The Author(s)

◀ **Figure EV3. Overview of condition-specific crosslinks.**

(**A**) Alignment of D1 (residues 1–212) and D2 (residues 205–447) between published crystal D1 and D2 dimer structures of PGL-1 (Aoki et al, 2016, 2021) and SWISS-MODEL(Waterhouse et al, 2018) structures of PGL-3. (**B**) Connectogram comparing crosslinking profiles of PGL-3 at 125 and 500 mM NaCl. Top: Shared between two conditions. Middle: Specific to 125 mM NaCl. Bottom: Specific to 500 mM NaCl.

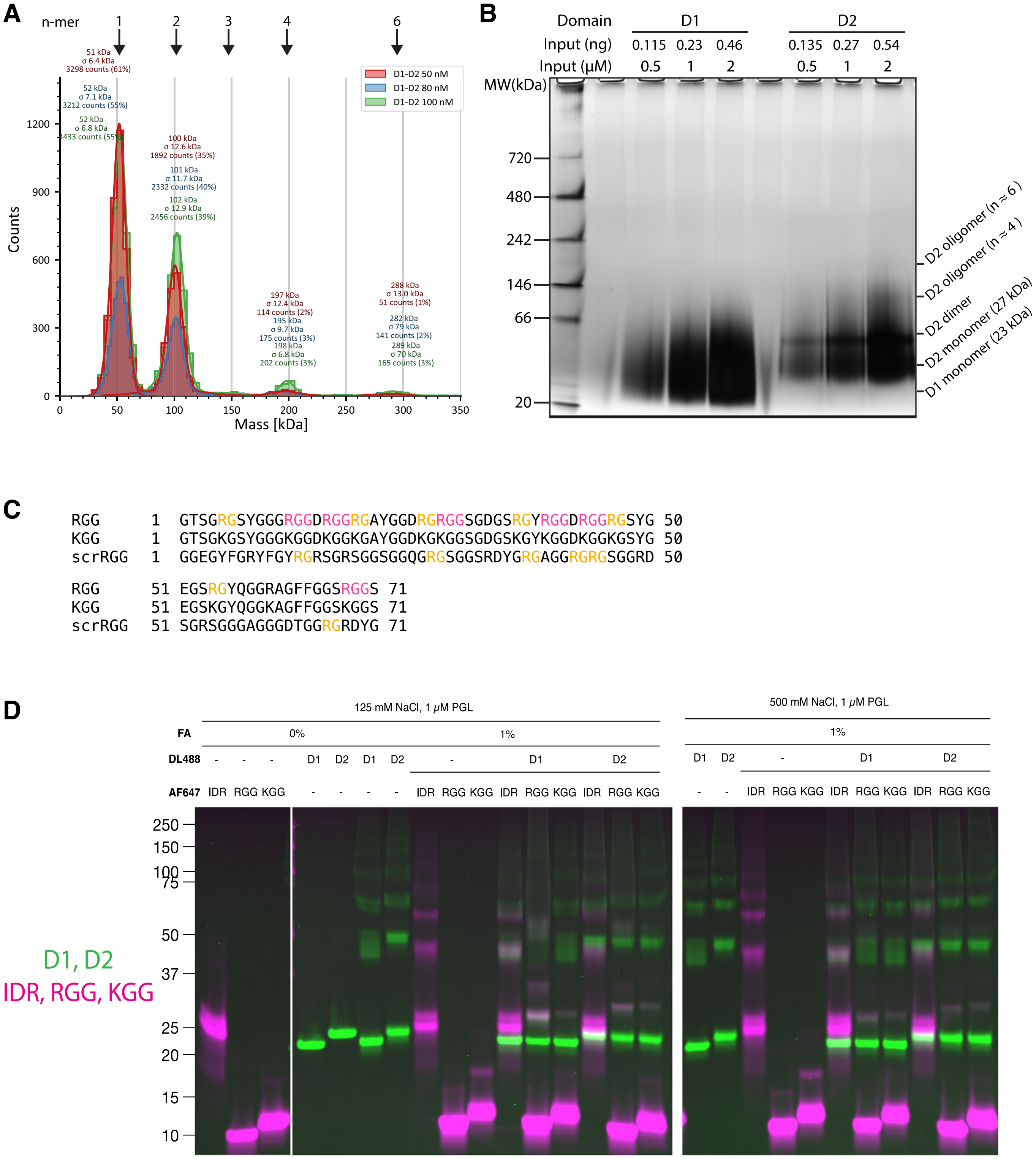

Figure EV4. PGL-3 D1 and D2 crosslinks to the RGG region.

(**A**) Mass histogram of D1-D2 oligomers in 125 mM NaCl solution, measured by mass photometry. D1-D2 was diluted by fourfold to reach the indicated concentrations in imaging wells and the videos were immediately recorded on a TwoMP (Refeyn). (**B**) Blue-native PAGE, visualized by silver stain, of single-domain constructs, running D1 and D2, of PGL-3 at indicated concentrations. (**C**) Sequence alignment among the native RGG peptide, the KGG peptide and the scrambled RGG peptide. RGG triplets and RG doublets are highlighted in magenta and yellow, respectively. (**D**) Dual-color fluorescent image of the gel shown in Fig. 5A. Two channels overlaid in color (DyLight 488 in green and Alexa Fluor 647 in magenta).

 © The Author(s)

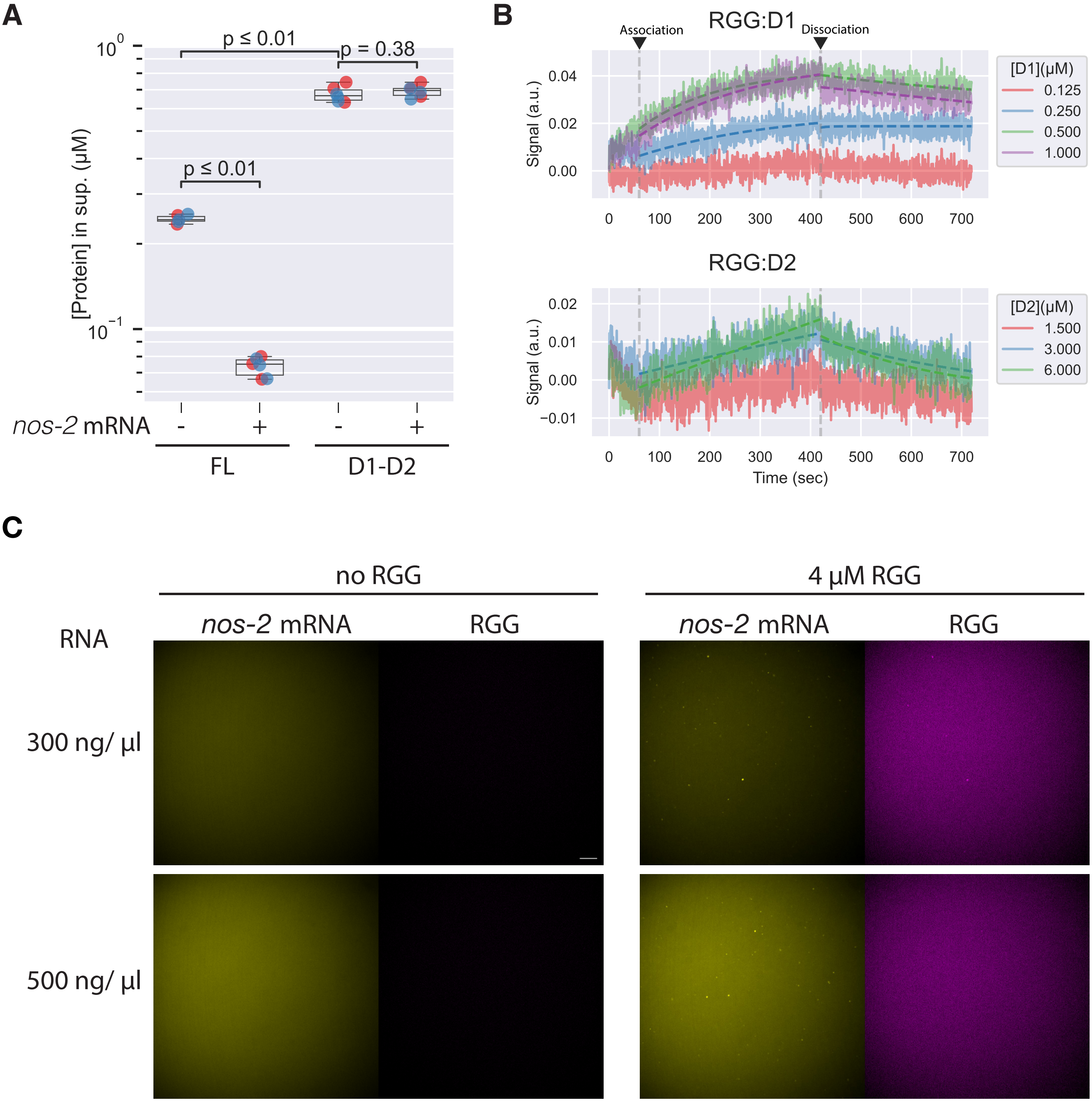

Figure EV5. Contributions of RGG domain to condensation.

(**A**) Plot showing dilute phase protein concentrations of condensation reactions carried out with 1.5 μM PGL-3, 125 mM NaCl in the presence or absence of 110 ng/μl *nos-2* mRNA. Different colors represent data from different days. In the overlaying box plots, lines inside boxes show median (Q2), box bounds are quartiles (Q1 and Q3), and whiskers show the range of data excluding outliers identified by the Turkey method. Technical replicates. *N* = 6. *P* values calculated by Wilcoxon rank-sum test with Benjamini–Hochberg adjustment for multiple comparisons. *P* values = 0.002165 (FL vs FL, RNA), 0.002165 (FL vs. D1-D2), 0.3768 (D1-D2 vs. D1-D2, RNA). (**B**) BLI sensorgrams for testing individual domains, D1 and D2, bind to the RGG peptide. At the indicated timepoints, the RGG peptide immobilized on the sensor tip was allowed to associate with indicated concentrations of D1 or D2. Dissociation steps were carried out in a buffer without D1 or D2. Note the differences in input concentrations between D1 and D2. (**C**) Fluorescence micrographs showing distribution of *nos-2* mRNA (yellow) and the RGG peptide (magenta) at indicated concentrations, without D1-D2. Note RNA and RGG form co-aggregates only when the two components are mixed. Scale bar = 10 μm.
